# Microbiomes of a disease-resistant genotype of *Acropora cervicornis* are resistant to acute, but not chronic, nutrient enrichment

**DOI:** 10.1038/s41598-023-30615-x

**Published:** 2023-03-03

**Authors:** J. Grace Klinges, Shalvi H. Patel, William C. Duke, Erinn M. Muller, Rebecca L. Vega Thurber

**Affiliations:** 1 Department of Microbiology, Oregon State University, 226 Nash Hall, Corvallis, OR 97331 USA; 2 Mote Marine Laboratory International Center for Coral Reef Research and Restoration, 24244 Overseas Hwy, Summerland Key, FL 33042 USA; 3 Mote Marine Laboratory, 1600 Ken Thompson Pkwy, Sarasota, FL 34236 USA

**Keywords:** Microbial ecology, Marine biology, Microbiome, Climate-change impacts

## Abstract

Chronically high levels of inorganic nutrients have been documented in Florida’s coral reefs and are linked to increased prevalence and severity of coral bleaching and disease. Naturally disease-resistant genotypes of the staghorn coral *Acropora cervicornis* are rare, and it is unknown whether prolonged exposure to acute or chronic high nutrient levels will reduce the disease tolerance of these genotypes. Recently, the relative abundance of the bacterial genus *Aquarickettsia* was identified as a significant indicator of disease susceptibility in *A. cervicornis*, and the abundance of this bacterial species was previously found to increase under chronic and acute nutrient enrichment. We therefore examined the impact of common constituents of nutrient pollution (phosphate, nitrate, and ammonium) on microbial community structure in a disease-resistant genotype with naturally low abundances of *Aquarickettsia.* We found that although this putative parasite responded positively to nutrient enrichment in a disease-resistant host, relative abundances remained low (< 0.5%). Further, while microbial diversity was not altered significantly after 3 weeks of nutrient enrichment, 6 weeks of enrichment was sufficient to shift microbiome diversity and composition. Coral growth rates were also reduced by 6 weeks of nitrate treatment compared to untreated conditions. Together these data suggest that the microbiomes of disease-resistant *A. cervicornis* may be initially resistant to shifts in microbial community structure, but succumb to compositional and diversity alterations after more sustained environmental pressure. As the maintenance of disease-resistant genotypes is critical for coral population management and restoration, a complete understanding of how these genotypes respond to environmental stressors is necessary to predict their longevity.

## Introduction

Populations of the Caribbean staghorn coral *Acropora cervicornis* have markedly declined since the 1980s due to a combination of stressors including infectious disease, poor water quality, high sea surface temperatures, and overfishing^1^. The decline of *Acropora cervicornis* has contributed to a reduction in shallow Caribbean coral reef cover from about 55% to less than 10% over the last 40 years^2,3^. To improve recovery of this species, asexual propagation and outplanting of *A. cervicornis* to in situ reef environments is employed in Florida’s coral reefs^4,5^ and throughout the greater Caribbean^6,7^. Outplanting of *A. cervicornis* demonstrably increases local coral coverage^8^, and nursery-grown corals may reach sexual maturity within 2 years after outplanting^4,8^. However, long-term survival of restored corals is predicted to be low if environmental stressors are not alleviated^9^. Outplanting efforts that do not consider integrating coral resistance and resilience to future stressors will therefore produce communities with low likelihood of future survival^10,11^.

A significant increase in nutrient concentrations due to fertilizer, top soil, and sewage runoff has been documented in the Florida Keys over the past three decades, with a peak in nutrient levels occurring around 2014^12,13^. The additive negative effect of nutrient enrichment and increased thermal stress leads to increased coral mortality^14–17^. A recent study indicated that acroporid species may be particularly susceptible to nutrient enrichment: nutrient-exposed *A. cervicornis* exhibited population mortality of 84–100% when exposed to a subsequent thermal stress event, while thermal responses of other coral species were not altered by nutrient exposure^14^. These impacts of nutrient exposure on coral health may be regulated by the coral holobiont: nitrogen enrichment leads to phosphate starvation in the symbiotic algae *Symbiodiniaceae*^18^, and disrupts nutrient cycling in coral-associated bacterial communities^19^. Nutrient enrichment can also increase microbial opportunism, decreasing microbial community richness and evenness, which may be tied to increased stressor-related mortality^16^. The impacts of nutrient enrichment on coral health, however, are not always negative and depend on nutrient source^20,21^, exposure time^22^, and ratios of available nitrogen to phosphorus^18,23^.

Across numerous studies, the coral microbiome has been identified as an important factor in both resistance to and recovery from environmental stressors^24–26^. It has been proposed that a highly diverse coral microbiome may provide a greater arsenal of antimicrobial defenses^27,28^ and that an evenly distributed, highly diverse microbiome may occlude niche space that could otherwise be filled by opportunistic bacterial species^28–30^. We previously found that microbiomes of *A. cervicornis* genotypes known to be disease-susceptible were characterized by an overwhelming dominance of the putative bacterial parasite “*Candidatus aquarickettsia rohweri*” (hereafter, *A. rohweri*), while disease-resistant genotypes were characterized by low relative abundance of* A. rohweri* and a more even and diverse microbiome^31–33^. We proposed that the high microbiome diversity of disease-resistant genotypes may provide greater antimicrobial defenses, and furthermore, that a coral host with low *A. rohweri* may have better immune capacity to combat future infections.

In contrast, dominance of a bacterial parasite such as that observed in disease-susceptible genotypes of *A. cervicornis* may pose a significant nutritional burden on the coral and lead to reduced or altered immune capacity. The dominance of *Aquarickettsia* in microbiomes of *A. cervicornis* has been observed across many studies and genotypes^34–37^ and a high abundance of its members is associated with increased disease prevalence, reduced coral growth, and increased tissue loss^16,38^. Despite the apparent disadvantages to high parasite abundance, *A. cervicornis* genotypes lacking this parasite are rare, suggesting that few genotypes are naturally resistant to *Aquarickettsia* infection^31,32^. As *Aquarickettsia* abundance responds positively to nutrient enrichment^33,38^, further exploration of the stability of identified genotypic associations of this parasitic genus is necessary considering the comparatively eutrophic conditions of Florida’s coral reef compared to other regions^12^.

Restoration efforts targeted towards coral genotypes with the highest stress resilience and long-term survival rates may increase the overall success of Caribbean coral restoration. Notably, disease-resistant genotypes are exceedingly rare in restoration broodstock, with only two genotypes from Mote Marine Laboratory’s collections in the Lower Keys exhibiting sustained disease resistance^39^ and comparably few resistant genets found in other Florida Keys nurseries^40^. Although disease-resistant genets of *A. cervicornis* possess diverse microbiomes with low abundances of the presumed parasite *Aquarickettsia*, a better understanding of the microbiome and phenotypic stability of these genets under shifting environmental regimes is necessary. We exposed fragments of the disease-resistant *A. cervicornis* genotype ML-7 to elevated nutrient levels to assess the individual and combined effects of ammonium, phosphate, and nitrate on *A. cervicornis* holobiont health, *Aquarickettsia* abundances, and changes in coral microbiome dynamics.

## Results

### Microbiomes of a disease-resistant *Acropora* genet are dominated by undescribed taxa

**Figure 1:**
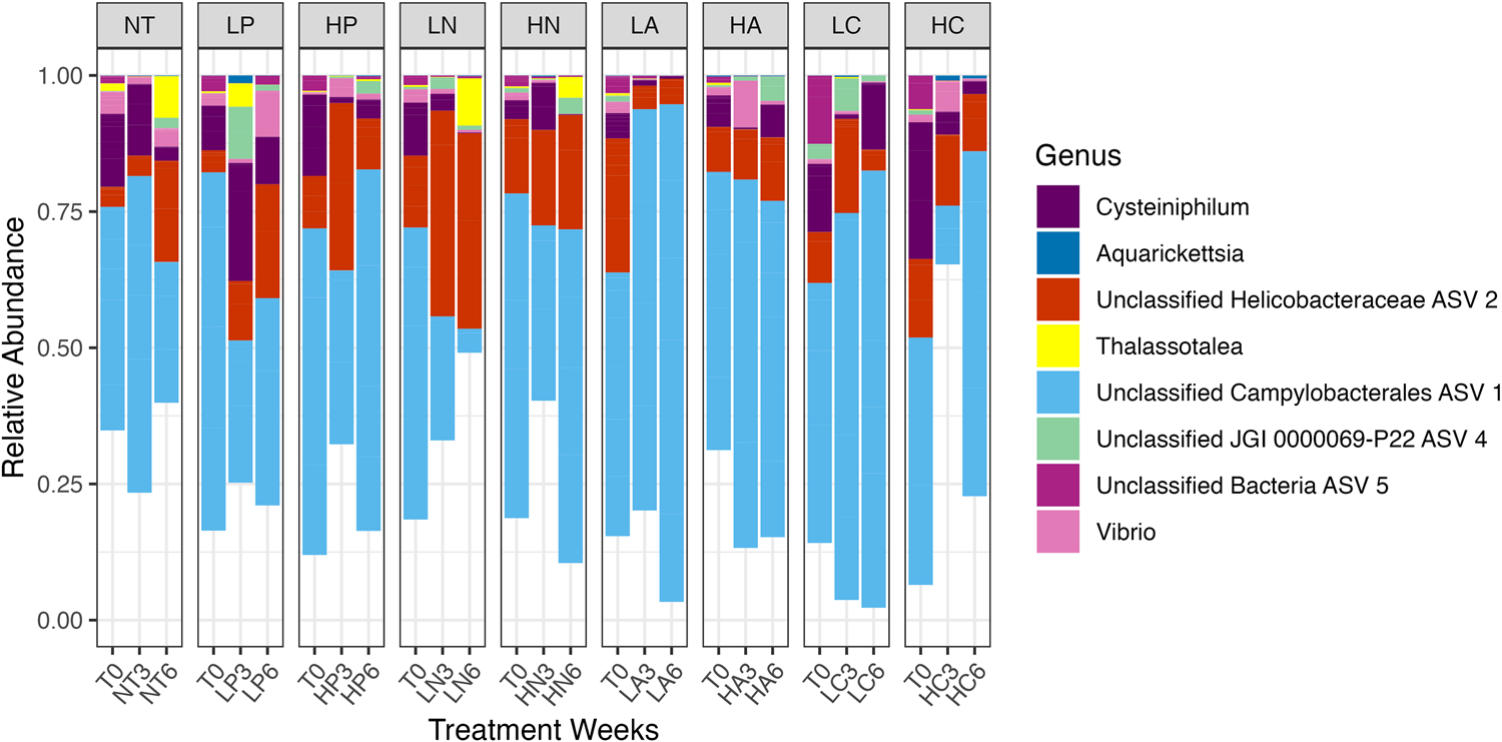
Relative abundance of the most abundant genera in genotype ML-7 across three time points in coral fragments exposed to different nutrient constituents. Taxa are included in the plot if they had a relative abundance greater than 1% across the entire dataset. The genus *Aquarickettsia*, though less than 1% relative abundance across the dataset, was included as this species has been found in high abundances of other genotypes of *A. cervicornis*. Each bar represents a minimum of 3 replicates. A total of 2598 ASVs of 680 genera are represented by this plot, including 672 genera that were less than 1% abundance across the dataset (uncolored portions of each bar). *NT* no treatment, *L* low (3× ambient), *H* high (4× ambient), *P* phosphate, *N* nitrate, *A* ammonium, *C* combined (P, N, A).

We exposed 154 ramets of the *Acropora cervicornis* genotype ML-7 to 6 weeks of nutrient enrichment in a controlled experimental setting. Corals were exposed to prolonged ammonium, nitrate, or phosphate enrichment, or to a combination of these three nutrients. Microbiomes of genotype ML-7 across all timepoints and treatments were dominated by a single undescribed bacterial species in the order Campylobacterales (Unclassified Campylobacterales ASV1). Mean relative abundance of this taxon across all samples was 50.77 ± 36.32% (Fig. 1). Abundance of this taxon was highly variable between samples: in some samples, Unclassified Campylobacterales ASV1 represented as much as 99.6%, while other samples completely lacked this sequence variant (Fig. 1). Unclassified Campylobacterales ASV1 was identified in 139 out of 154 total samples. Other abundant taxa included a single unclassified bacterial species from the family *Helicobacteraceae* (12.70 ± 21.86%, max of 95.54%, present in 106/154 samples) and a species from the genus *Cysteiniphilum* (6.05 ± 12.76%, max of 93.76% and present in 139/154 samples) (Fig. 1). A different ASV also identified as the genus *Cysteiniphilum* was less abundant and common (0.550 ± 4.423% and present only in 8 samples) but reached a maximum of 53.02% in one sample (Fig. 1). An ASV classified as the genus *Thalassotalea* was variably abundant across the dataset, with a low mean relative abundance of 0.858 ± 5.010%, yet reaching a maximum of 51.41% in one sample. Confirming previous results, an ASV identified as *Aquarickettsia rohweri*, the presumed parasite of disease-susceptible *Acropora* genotypes, was in very low abundance across samples of the disease-resistant genotype ML-7, with a mean relative abundance of 0.494 ± 3.141% (Fig. 1). Only one sequence variant in the genus *Aquarickettsia* was found across all samples.

The top four BLASTn results for the abundant unclassified Campylobacterales ASV1 were to unclassified taxa from samples previously collected from *Acropora cervicornis* or *A. palmata,* with percent identity ranging from 100 to 98.42% (EU861195, GU118068, GU117990, GU118110^41^). All other BLAST results had a percent identity below 90% and were classified as *Sulfurimonas* or other Campylobacterota. The sequence variant unclassified past *Helicobacteraceae* (ASV2, also a member of Campylobacterales) was identical to two sequences (GU117961 and GU117962) from *Acropora cervicornis* samples in Panama^41^ as well as a sequence from the Caribbean coral *Montastraea annularis* exhibiting signs of white plague (AF544892^42^). While these two highly abundant ASVs were both members of Campylobacterales, they shared fairly low homology (83.1%). However, the 16S rRNA sequence of Unclassified Campylobacterales ASV1 was highly similar to that of another abundant unclassified Campylobacterales, ASV9 (99.6% similar). The ASV identified as *Thalassotalea* was identical to *Thalassotalea euphylliae* strain Eup-16 (NR_153727), isolated from the coral *Euphyllia glabrescens*^43^, as well as to other sequences identified as *Thalassotalea* and found in the corals *Galaxea fascicularis* and *Acropora millepora* (KU354186, MW828542).

### Microbiomes of a disease-resistant *Acropora* genet only exhibited changes after several weeks of exposure to tank conditions

**Figure 2:**
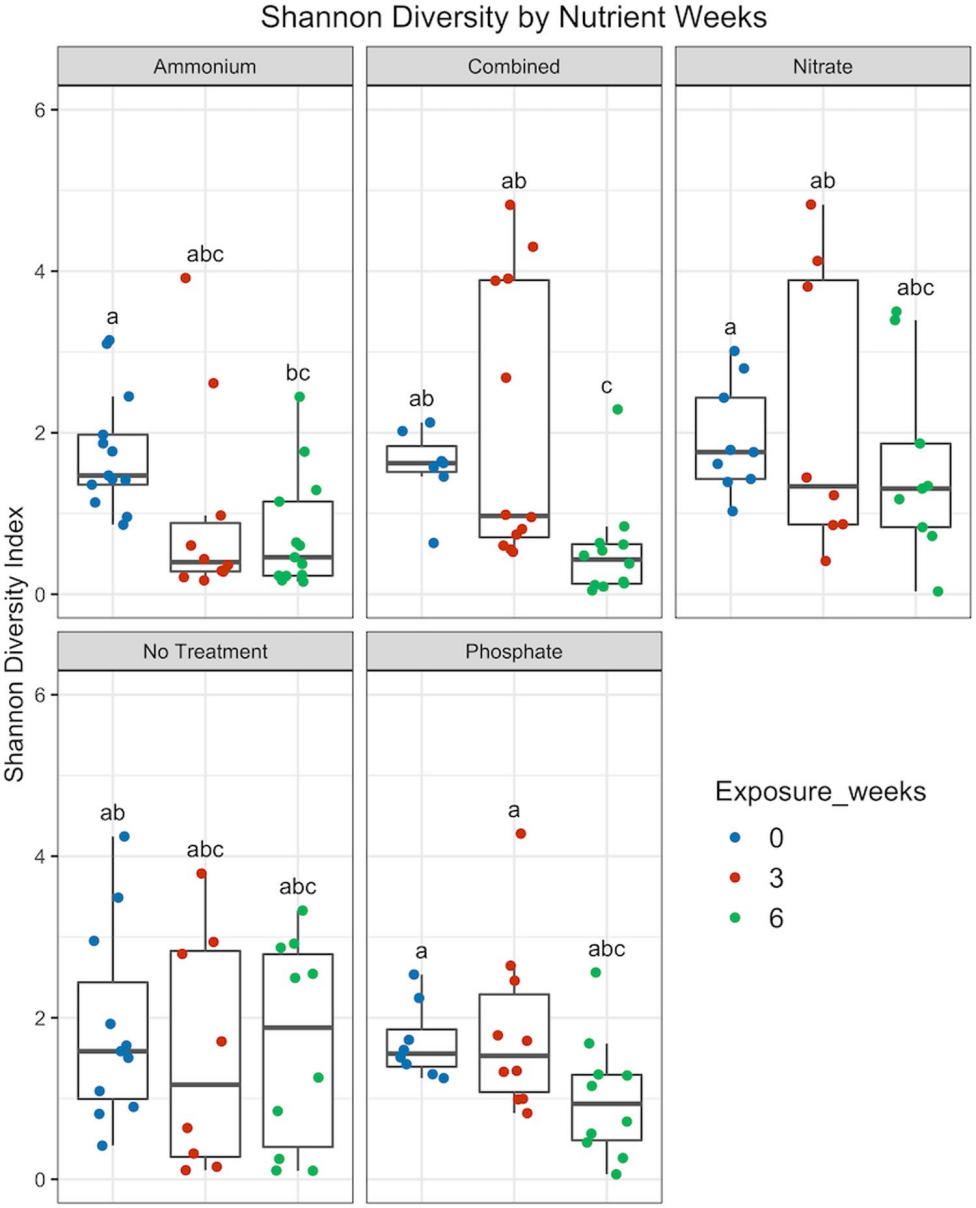
Differences in Shannon diversity by treatment (Ammonium, Combined, Nitrate, Phosphate, or no treatment) and exposure weeks (0, 3, and 6) in genotype ML-7. Boxes sharing a letter are not significantly different from each other using an FDR corrected significance level of *p* < 0.05.

Analyses of Shannon diversity indicated that coral fragments exposed to ammonium or a combination of nutrients (ammonium, phosphate, and nitrate) had significantly lower community diversity by week 6 than samples at week 0 (Fig. 2, *p* < 0.05, χ^2^ = 32.122). No individual treatment experienced a significant decline between week 0 and week 3 of the experiment (Fig. 2, *p* > 0.05, Supp. Table 3). However, there was a significant decrease in overall species richness and evenness in nutrient-treated samples between weeks 0 and 6 and between weeks 3 and 6 (*p* < 0.001, χ^2^ = 24.254, Supp. Fig. 3). There were no significant differences in Shannon diversity in untreated samples over the course of the experiment (Supp. Fig. 3, Supp. Table 3).

**Figure 3:**
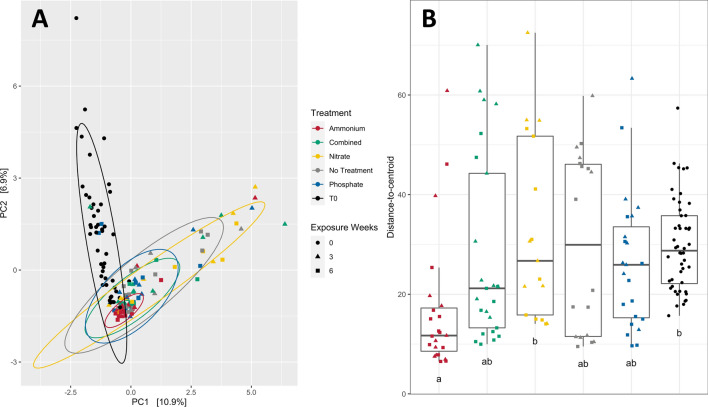
(**A**) Principal components analysis ordination of genotype ML-7 samples using Euclidean distance on centered log ratio-transformed data, colored by nutrient treatment. (**B**) Differences in dispersion, as distance-to-centroid, by treatment (ammonium, combined, nitrate, phosphate, or no treatment). Boxes sharing a letter are not significantly different from each other using an FDR corrected significance level of *p* < 0.05. Note that the two panels share a legend (center).

Shifts in microbial community composition (i.e., beta diversity) likewise occurred over the course of the experiment, but were primarily a factor of time in the aquarium system, rather than exposure to particular nutrient constituents. Principal components analysis was performed on samples from all timepoints and treatments using CLR-transformed data and Euclidean distance to compare dissimilarity (Fig. 3A). Samples from time point zero (not yet exposed to treatments or tank conditions) clustered separately from later timepoints, and there was no visible distinction between treatments at 3 and 6 weeks in the PCA. Pairwise comparisons by nutrient exposure weeks (df = 10, F = 1.9617) indicated that all treatments at weeks 3 and 6 were distinct from communities at T0 (pairwise PERMANOVA with FDR correction, *p* < 0.05, Supp. Table 4). Ammonium-treated samples showed a significant difference in beta diversity compared to both nitrate-treated and untreated samples by week 6 of the experiment (*p* = 0.029 and *p* = 0.005, Supp. Table D4), and these samples notably exhibited much tighter clustering in PCA ordination than other treatments (Fig. 3A). Nitrate- and phosphate-treated samples were significantly different from each other by week 6 (*p* = 0.039, Supp. Table 4). All other treatments were not distinct within timepoints at α = 0.05 (Supp. Table 4). Differences in beta diversity between high and low levels of nutrient enrichment across all treatments were not significant, though both levels were significantly different from controls (pairwise PERMANOVA with FDR correction, *p* < 0.01; Supp. Table 5).

Tests for differences in dispersion (within-group variation) showed that sample-to-sample variation was affected by exposure duration (F = 5.4924, *p* = 0.005) and nutrient type (F = 2.6867, *p* = 0.009). Dispersion significantly decreased between T0 and week 6 (pairwise PERMDISP, *p* = 0.012) and between weeks 3 and 6 (*p* = 0.024), but did not decrease between T0 and week 3 (*p* = 0.713). Pairwise tests for differences in group dispersion by nutrient treatment showed that microbiomes of ammonium-treated samples (weeks 3 and 6) had significantly lower dispersion than nitrate-treated and T0 samples (Fig. 3B, p < 0.05, Supp. Table 6). When community dispersion was examined by nutrient treatment and exposure weeks, dispersion decreased significantly over time in only ammonium-treated samples (Supp. Fig. 4, *p* = 0.035).

### Numerous microbial taxa responded to nutrient enrichment

**Figure 4:**
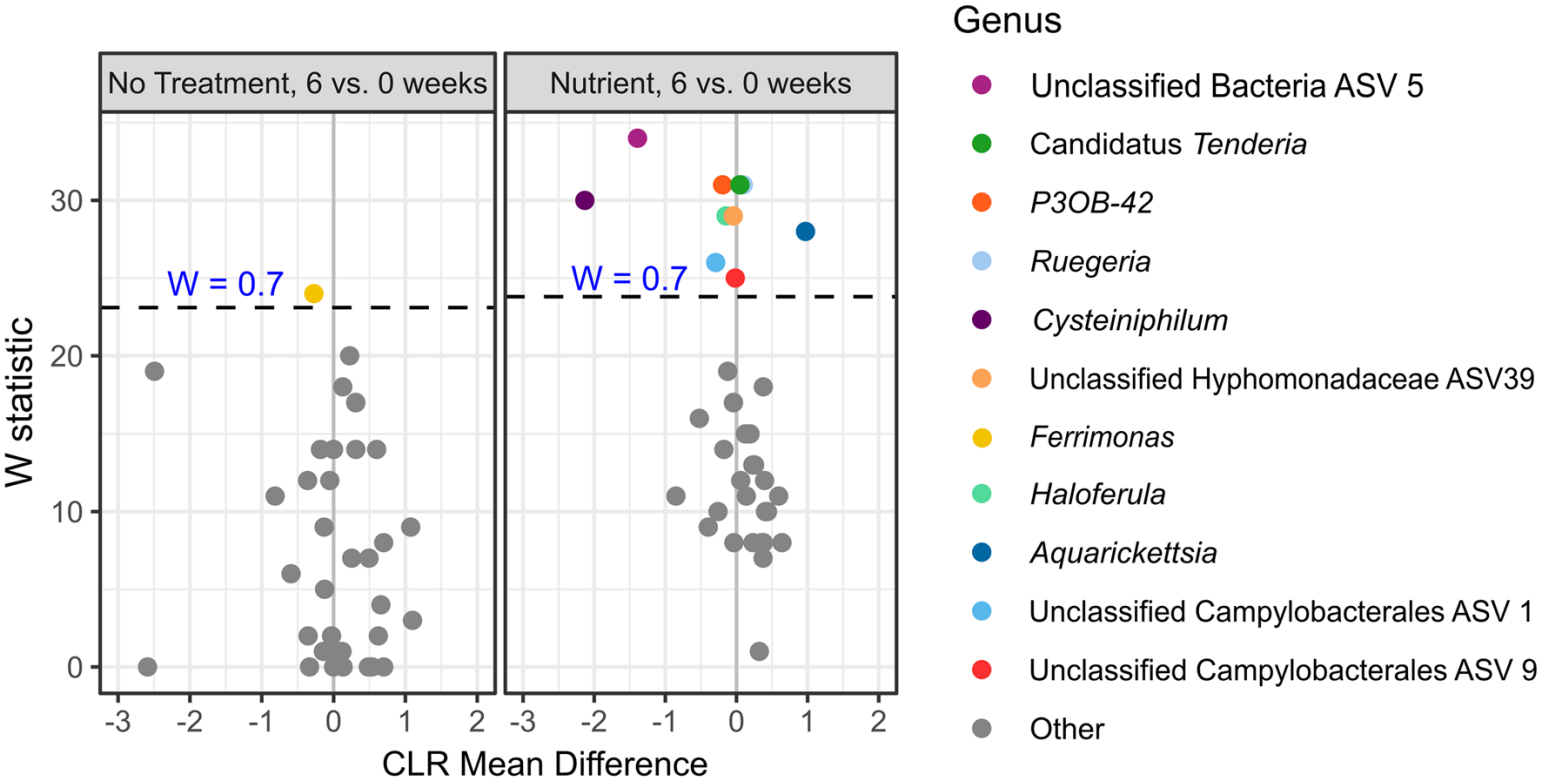
Volcano plot of results from differential abundance analysis with ANCOM-II on genotype ML-7 samples by treatment condition at 6 weeks vs 0 weeks. Data were subset to either only no treatment samples, or all other treatments pooled together (“nutrient”). The W statistic represents the strength of the test for the 35 tested species. Taxa above the dashed line are significant with the null-hypothesis rejected 70% of the time (W = 0.7). Non-significant taxa are colored grey. The x-axis value presents the effect size as the CLR (centered log ratio)-transformed mean difference in abundance of a given species between the two groups being compared. A positive x-axis value indicates that a genus was more abundant at 6 weeks than 0 weeks (abundance increased with time).

Shifts in individual taxa over the course of the experiment were examined as differential abundance with the tool ‘ANCOM-II’ (Fig. 4, Supp. Fig. 5). ANCOM-II presents an analysis of changes in taxa(on) relative abundance in comparison to the geometric mean of taxa in a given group, testing the null hypothesis that the average abundance of a given species in a group is equal to that in the other group. Although corals were allowed two weeks to acclimate to experimental tank conditions prior to experimentation, one taxon, *Ferrimonas futtsuensis*^44^, declined in abundance in untreated fragments over the course of 6 weeks, though the magnitude of change was small (Fig. 4, Supp. Fig. 5A). Furthermore, this taxon was low abundance across the dataset, averaging 0.167 ± 0.776%. In contrast, exposure to nutrient treatment was associated with shifts in numerous taxa over the course of the experiment. Analyses of all nutrient treatments pooled together identified nine taxa that responded significantly to nutrient-treated conditions (Fig. 4, Supp. Fig. 5B). Despite the overall low abundance of reads mapping to the parasitic genus *Aquarickettsia* in genotype ML-7 samples (0.494 ± 3.141%), this taxon increased significantly by 6 weeks of nutrient enrichment compared to T0. Relative abundance of this taxa was only 0.021 ± 0.101% at week 0 and increased to 0.143 ± 2.67% at week 6 in nutrient-enriched samples, equating to an increase of about sevenfold. An ASV within the genus *Ruegeria* was also significantly more abundant in nutrient-enriched samples, though fold change was low. Unclassified Campylobacterales ASV1 exhibited a significant decline in abundance across nutrient-treated samples. An ASV in the genus *Cysteiniphilum* also significantly declined with nutrient exposure, with a negative response of more than twofold. Unclassified Bacteria ASV5 (with closest BLAST homology to Deltaproteobacteria) declined with nutrient exposure with a negative CLR-fold change of 1.4.

**Figure 5:**
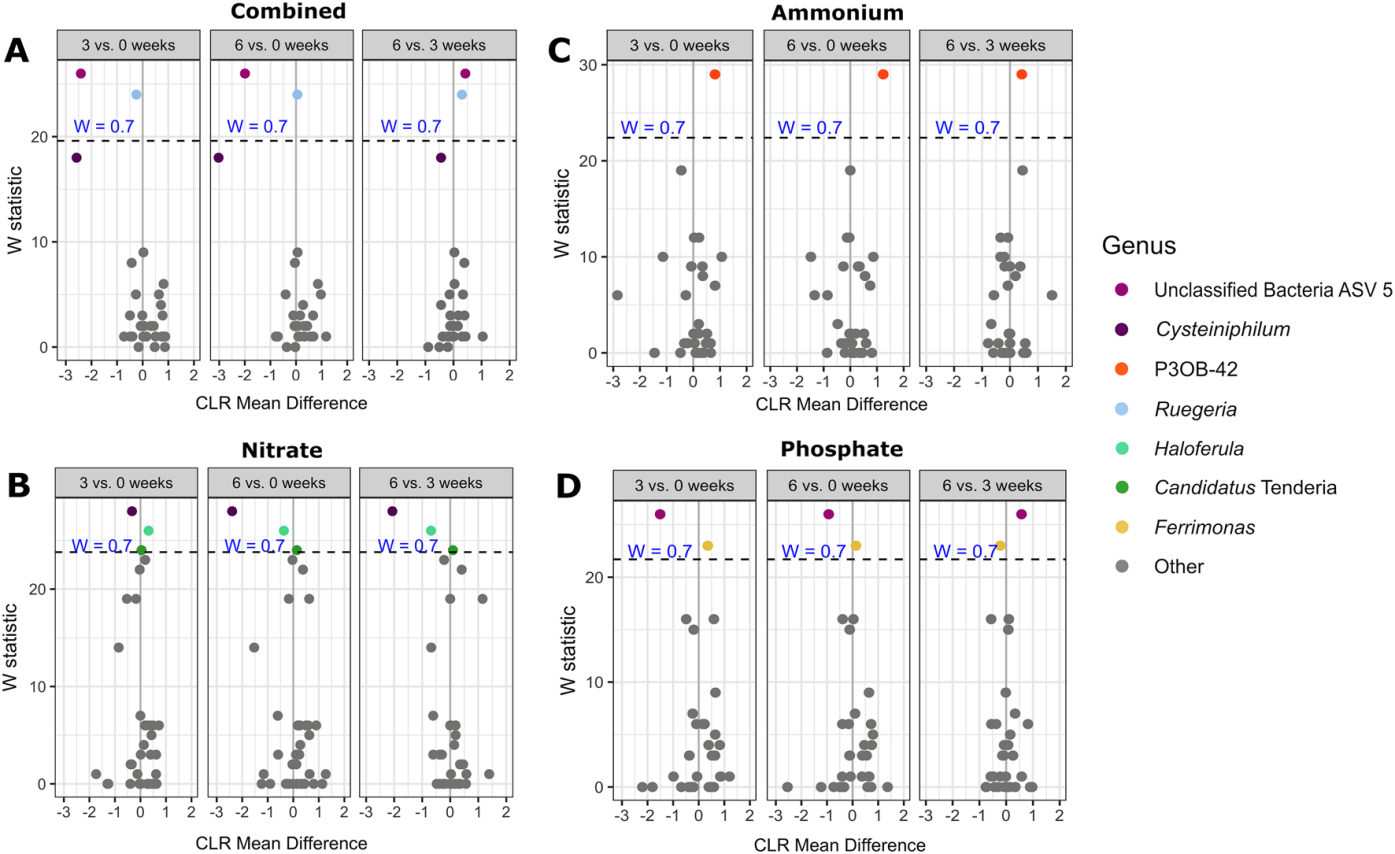
Volcano plot of results from differential abundance analysis with ANCOM-II on genotype ML-7 samples by individual treatment condition, for 3 vs 0 weeks, 6 vs 0 weeks, and 6 vs 3 weeks. Data were subset to examine each nutrient treatment individually. (**A**) Combined treatment. (**B**) Nitrate treatment. (**C**) Ammonium treatment. (**D**) Phosphate treatment. For the first panel of each nutrient, a positive x-axis value means the genus was more abundant in combined-treated samples at 3 weeks compared to 0 weeks (abundance increased with time).

When ANCOM was performed as comparisons isolating each nutrient treatment individually, no individual treatment induced an increase in the genus *Aquarickettsia.* Combined treatment (nitrate, phosphate, and ammonium) induced a significant decrease in Unclassified Bacteria ASV5 and a slight increase in an ASV from the genus *Ruegeria.* Changes in the genus *Cysteiniphilum* did not surpass significance criteria of W = 0.7 (null hypothesis rejected 70% of the time), but did surpass the default ANCOM significant criterion of W = 0.6, exhibiting a ~ threefold reduction by 6 weeks of exposure to combined treatment compared to T0 (Fig. 5A). Nitrate treatment induced a significant reduction in *Cysteiniphilum* and an initial increase in the genus *Haloferula*, which then declined between 3 and 6 weeks of nitrate enrichment (Fig. 5B). Only an ASV belonging to the genus *P30B-42* changed significantly with ammonium treatment, despite significant changes in community structure as indicated by alpha and beta diversity metrics in this treatment (Fig. 5C). Phosphate treatment induced a significant reduction of Unclassified Bacteria ASV5 and an increase in *Ferrimonas futtsuensis* (Fig. 5D)*.*

### Nitrate treatment led to reduction of coral host linear extension

Although microbial community structure was not strongly impacted by nitrate enrichment, chronic exposure to enriched nitrate levels led to a reduction in coral fragment linear extension. Differences in total linear extension (TLE, n = 107) were not significant by treatment at 3 weeks of exposure, but were significant by 6 weeks of exposure (Supp. Fig. 6, Supp. Table 7). Corals exposed to no treatment grew an average of 6.45 ± 1.81 mm by 6 weeks (n = 11), while fragments exposed to 3× ambient levels of ammonium grew the most of any coral fragments across treatments, at 7.00 ± 1.87 mm of growth (n = 6) by 6 weeks. Exposure to 4× ambient concentrations of nitrate treatment resulted in the lowest amount of TLE over the course of the experiment (3.33 ± 1.97 mm, n = 6). TLE of nitrate-enriched corals was significantly lower at 6 weeks compared to 3× ambient ammonium- and 3× phosphate-treated corals, and to untreated corals (*p* < 0.05, Tukey’s pairwise honest significance test on log-transformed data). Corals had significantly greater lengths at week 6 compared to week 3 for all treatments except for nitrate (Supp. Fig. 6).

Symbiont densities as visually assessed using a CoralWatch Health Chart responded positively to tank conditions, with chart scores (scale of 1–6) increasing from an average of 4.86 ± 0.528 (n = 55) at week 0 to 5.90 ± 0.300 by week 6 (n = 61). All corals exhibited increases in symbiont density and differences by treatment were insignificant (*p* > 0.05, Kruskal–Wallis test with Benjamini–Hochberg correction), although average health scores for untreated corals at 6 weeks (5.82 ± 0.405, n = 11) trended lower than for nutrient-enriched corals (5.92 ± 0.272, n = 50, 3× and 4× samples of all nutrient treatments).

### Microbiomes of genotypes ML-7 and ML-50 are distinct and remain distinct with nutrient exposure

**Figure 6:**
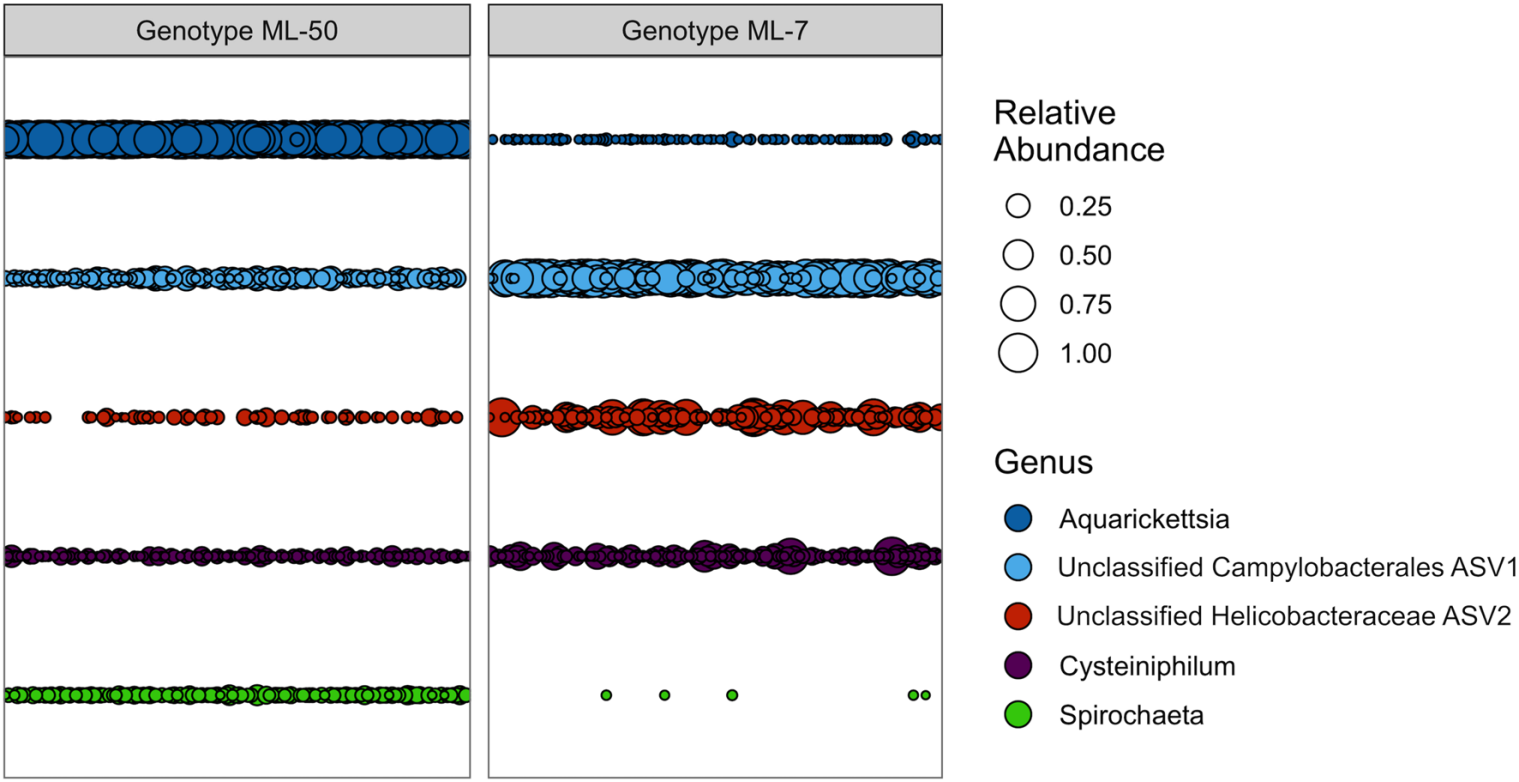
Relative abundance of most abundant genera (> 1.5% relative abundance) between two genotypes of *Acropora cervicornis* (n = 150 samples per genotype) exhibiting differing disease susceptibility (genotype 7 and genotype 50).

To test whether the microbiota of disease-resistant and -susceptible *A. cervicornis* genotypes respond differently to nutrient exposure, we compared microbiome data from disease-resistant genotype ML-7 (the present study) to our previously published data on disease-susceptible genotype ML-50, exposed to the exact same nutrient enrichment regime at the same time^33^ but in different aquaria to prevent potential bacterial transmission between the coral genotypes. Several taxa were common to the two genotypes, though relative abundances of these taxa differed (Fig. 6). *Aquarickettsia* was dominant across all samples of genotype ML-50 (82.72 ± 13.94%^33^), with a relative abundance approximately 167 times that in genotype ML-7, in which it averaged 0.494 ± 3.141%. Genotype ML-7 microbiomes were instead dominated by Unclassified Campylobacterales ASV 1 (50.77 ± 36.32%). This ASV was also common but not ubiquitous (116/150 samples) and in lower abundance (6.67 ± 7.88%) in genotype ML-50 samples (roughly 7.6× higher abundance in genotype ML-7 than ML-50). An ASV identified as a member of the family *Helicobacteraceae* was likewise in high abundance in genotype ML-7 (12.70 ± 21.86%) and present in some samples (56/150) of genotype ML-50 (0.87 ± 1.95%). An ASV mapping to the genus *Cysteiniphilum* was present in both genotypes (genotype ML-7: 6.05 ± 12.76%, genotype ML-50: 1.73 ± 3.35%). An ASV mapping to the genus *Spirochaeta* was almost exclusive to genotype ML-50, though consistently at low abundance (3.87 ± 3.02%, present in 128/150 samples). This genus was found only in 5 samples of genotype ML-7, with an average abundance of 7.66e^-5^ ± 0.0005%.

**Figure 7:**
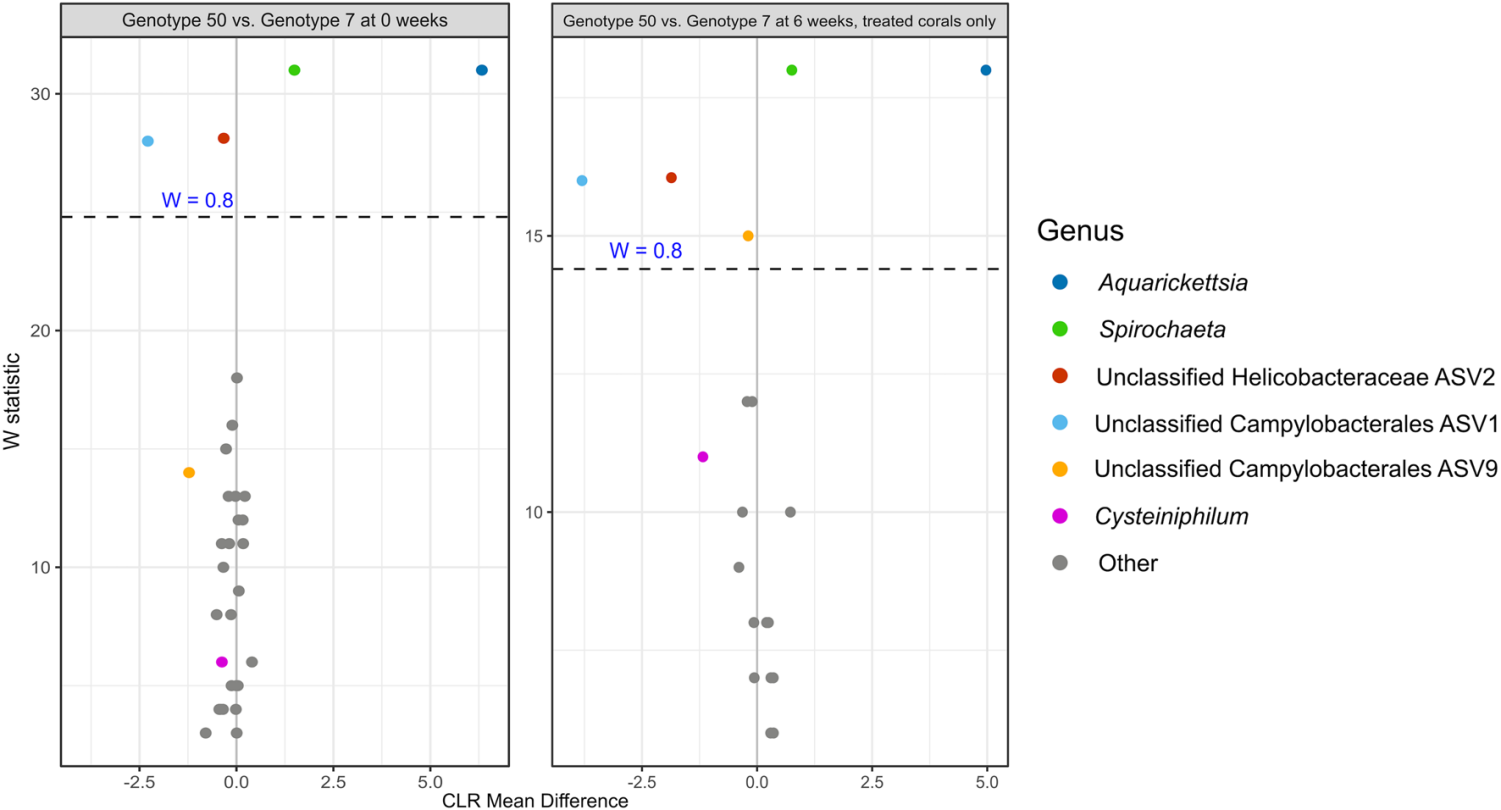
Volcano plots of results from differential abundance analysis with ANCOM-II, comparing genotype 7 and genotype 50 at two timepoints. Contrasts were performed within the full ASV table subset to by timepoint. The W statistic represents the strength of the test for the 35 tested species. Taxa above the dashed line are significant with the null-hypothesis rejected 80% of the time (W = 0.7). Non-significant taxa are colored grey, with the exception of the genus *Cysteiniphilum*, which was highly abundant in both genotypes and therefore of interest for comparison. A positive x-axis value indicates the genus is more abundant in genotype 50 samples compared to genotype 7 or vice versa for a negative x-axis value.

To assess differential responses of the two genotypes to nutrient stress, we compared microbiomes of both genotypes from T0 (untreated conditions, prior to experimental manipulation) to microbiomes after 6 weeks of nutrient exposure. Genotype ML-7 harbored significantly higher alpha diversity than genotype ML-50 both prior to nutrient enrichment and after 6 weeks in the experimental system, regardless of treatment (Supp. Fig. 7, *p* ≤ 0.001, Shannon χ^2^ = 88.638), though both genotypes decreased in overall alpha diversity over the course of the experiment (Supp. Fig. 7). To examine response of the two genotypes to nutrient enrichment, ANCOM was used to identify differentially abundant taxa for each genotype at timepoint zero (no nutrient exposure) and post exposure to nutrient treatment (week 6, all treated corals) (Fig. 7). ASVs corresponding to *Aquarickettsia* and *Spirochaeta* were significantly more abundant in genotype ML-50 than in genotype ML-7 at both 0 and 6 weeks of experimentation, while Unclassified Campylobacterales ASV1 and Unclassified Campylobacterales ASV13 (one nucleotide difference from ASV1) were more abundant in genotype ML-7 at T0. After 6 weeks of any nutrient treatment, the ASV from the family *Helicobacteraceae* was significantly greater in abundance in genotype ML-7 than in genotype ML-50. The genus *Cysteiniphilum*, which was moderately abundant in both genotypes, was not significantly differentially abundant by genotype at either timepoint.

Microbial beta diversity of the two genotypes was significantly different throughout the course of the experiment in nutrient-treated samples (Supp. Fig. 8A, *p* < 0.01, R^2^ = 0.0712). Despite the greater similarity of T0 samples across both genotypes in PCA ordination compared to later timepoints (Supp. Fig. 8B), pairwise comparisons indicated that beta diversity between the two genotypes was significantly different at T0 (*p* < 0.01, R^2^ = 0.0827), as well as all subsequent timepoints in treated samples (Supp. Table 8). Bacterial community dispersion changed significantly over time in both genotypes, declining by 6 weeks of nutrient exposure (pairwise PERMDISP with FDR correction, F = 30.86, Supp. Fig. 9). Genotype ML-50 reached a new stable state of low community dispersion by only 3 weeks of exposure, while significant changes in dispersion were not documented in genotype ML-7 until week 6 of the experiment (Supp. Table 9). Genotype ML-7 had significantly higher dispersion than genotype ML-50 at all three timepoints (Supp. Table 9).

## Discussion

### The role of microbial diversity in the face of environmental stressors

The coral microbiome likely plays a significant role in both resistance to and recovery from stressors. As bacterial symbionts of coral are proposed to perform numerous functions including pathogen defense, nitrogen and sulfur cycling, and nutrient translocation to the host, a diverse microbiome containing organisms fulfilling each of these services may allow for optimal holobiont response to environmental shifts^27,28^. While microbiome responses to short-term stressors (such as thermal stress) may be reversed with the removal of the stressor^45–47^, cumulative and long-term stressors (including nutrient enrichment) can shift the coral microbiome from mutualistic to pathogenic^24,48^. In this study, microbiome community structure in genotype ML-7 of the coral *Acropora cervicornis* (previously characterized as disease-resistant^39^) was not altered by short-term exposure (i.e., 3 weeks) to significantly elevated levels of nutrient enrichment. There were no significant changes in diversity and few taxa responded to 3 weeks of nutrient enrichment, suggesting that microbial communities of genotype ML-7 samples were initially resistant to nutrient exposure and did not experience community shifts. Chronic (6 weeks) exposure to nutrients, however, led to changes in microbiome diversity and variability, with a significant decrease in overall species richness and evenness. We additionally found that although genotype ML-7 harbored the putative coral parasite, genus *Aquarickettsia*^49^, relative abundances remained low or undetectable in many samples even after prolonged nutrient enrichment, which increased abundances of this genus in other studies^16,33,38^.

Impacts of nitrate on corals in this study may have been compounded by high ambient levels of nitrate in untreated aquarium source water in comparison to Looe Key Reef (Supp. Fig. 1). It is therefore surprising that this treatment had minimal impact on microbial community structure compared to other treatments. Nitrate enrichment did, however, demonstrably impact growth rates, with 6 weeks of nitrate enrichment significantly reducing growth rates compared to untreated corals while no other group responded significantly. The reduction of growth with nitrate enrichment is consistent with previous findings that nitrogen enrichment leads to an overgrowth of the algal symbiont *Symbiodiniaceae*, which subsequently fixes carbon so rapidly that coral calcification rates are limited^20,50^. In a previous study, we found that phosphate, rather than nitrate, reduced growth rates of genotype ML-50, which we hypothesized was linked to the positive response of the putative parasite *Aquarickettsia* to phosphate enrichment^33^.

### Potential roles of dominant bacterial taxa from the order Campylobacterales

Microbiomes of genotype ML-7 were dominated by two sequence variants classified as Campylobacterales, and both of these ASVs were also found in samples of Panamanian *A. cervicornis*^41^. The dominance of two taxa classified as Campylobacterales in samples from both Florida and Panama merit further study of the relationship of this order with Acroporid species. Furthermore, a sequence variant identical to ASV1 was found in high abundance in genotype ML-7 as well as in several other genotypes sourced from throughout the Florida Keys sampled in 2019 in Ref.^32^. These unclassified Campylobacterales were not found in high abundance in samples of genotype ML-7 taken from Mote’s in situ nursery in 2015^31^, which suggests that the dominance of these taxa may be a recent development. The order Campylobacterales is primarily comprised of microaerobic chemolithotrophic species, including many that oxidize sulfur^51,52^. Sequence variants from Campylobacterales have been found in association with Stony Coral Tissue Loss Disease (SCTLD) and may thrive on decaying and anoxic tissue^53^. These SCTLD-associated taxa, however, were from the genus *Sulfurimonas* and the family *Arcobacteraceae*^53^*,* while the taxa from our study (Unclassified Campylobacterales ASVs 1 and 9) were not closely related to any named group in Campylobacterales. It is therefore difficult to predict the function of the unclassified Campylobacterales harbored by genotype ML-7, though the absence of tissue necrosis suggests that these taxa are more likely inhabiting a different ecological niche than those associated with SCTLD. The methodology of the present study included the sampling of coral skeleton, tissue, and mucus simultaneously. As coral skeleton has been previously found to harbor anaerobic bacteria^54,55^, it is possible that bacterial biomass in this genotype is relatively low in the mucus and tissue layers, such that overall microbial community composition is dominated by microaerobic bacterial living in skeletal niches. Anaerobic sulfur-oxidizing species have been found in high abundance in skeletons of other coral genera and have been proposed to play a beneficial role by detoxifying sulfide and supplying their host with organic nutrients^55,56^. Further study of genotypes dominated by these unclassified Campylobacterales should aim to identify metabolic pathways enriched in these samples and determine whether these taxa are most dominant in skeletal compartments.

### Chronic ammonium exposure led to decreased microbiome diversity in genotype ML-7 corals

Ammonium is often naturally-derived in reef systems from fish excretion and enhances coral growth^20,57^. In this study, ammonium treatment did not significantly increase coral growth compared to untreated conditions, though mean linear extension at 6 weeks was higher for corals treated with ammonium than any other treatment. Shannon diversity declined in response to ammonium and combined (N, P, and A) treatment but no single taxon decreased significantly in ammonium-treated samples. The loss of minor taxa that varied by individual fragment may therefore lead to declines in alpha diversity with ammonium treatment. Indeed, mean per-sample species richness of ammonium-treated samples halved between 0 and 6 weeks of exposure, decreasing from 505 taxa to 250. Ammonium treatment significantly altered beta diversity compared to untreated samples by 6 weeks of nutrient enrichment, and dispersion of ammonia-treated samples decreased significantly over time while no other treatment led to reduced dispersion. The reduced diversity and dispersion of ammonium-treated samples at the conclusion of the experiment may reflect the loss of rare taxa as a result of increased competition from dominant taxa (Unclassified Campylobacterales ASV1, Unclassified *Helicobacteraceae* ASV2, *Cysteiniphilum*). Interestingly, ammonium treatment also induced the proliferation of family P30B-42, a member of Myxococcales, which has been suggested to play a commensal or beneficial role based on its relatively high abundances in disease-resistant genotypes of *Acropora*^34^. Notably, significant changes in overall microbial community structure and diversity were not significant until 6 weeks of ammonium exposure, suggesting that microbiomes of genotype ML-7 may withstand short-term environmental shifts without alteration in structure, but long-term exposure to elevated nutrients may stimulate the growth of certain microbial taxa and/or the loss of rare taxa.

### Individual taxa responded to nutrient enrichment without significantly altering microbiome structure

While ammonium enrichment alone appeared to reduce rare microbial taxa and decrease community dispersion, other forms of nutrient enrichment, as well as ammonium combined with other forms of enrichment, reduced dominant taxa and stimulated rare taxa. While the dominant taxa genus *Cysteiniphilum* and Unclassified Bacteria ASV5 declined in response to nutrient treatment, the genus *Aquarickettsia* increased significantly in these samples, despite a low average abundance of this genus in genotype ML-7 samples (0.191 ± 0.842%). The positive response of *Aquarickettsia* to nutrient-enriched conditions has been previously documented^33,38^. The increase in *Aquarickettsia* in samples of genotype ML-7 is notable as disease-susceptible genotypes of *A. cervicornis* are characterized by high abundance of this putative parasite^31^. Although relative abundance remained low across the experiment, the increase over the course of the experiment was considerable, increasing from 0.021 ± 0.101% at week 0 to 0.143 ± 2.67% at week 6 in nutrient-enriched samples, equating to an increase of about sevenfold. Individual nutrient treatments did not increase the abundance of *Aquarickettsia* in samples from this study, though phosphate enrichment had been previously found to stimulate abundances of this putative parasite^33^. The lack of clear response of this genus to a single treatment, despite a statistically significant increase in nutrient-treated samples as a group compared to untreated samples, suggests that changes in this taxon may have been stochastic and highly dependent on abundance of this taxon at the start of enrichment: only 29 of 48 timepoint 0 samples had detectable *Aquarickettsia*.

The observed loss of *Cysteiniphilum* in response to nutrient treatment may be a result of its strictly aerobic respiration: nutrient enrichment can induce localized hypoxic conditions that may affect bacteria living in coral mucus or tissue^58^. Unclassified Bacteria ASV5 responded significantly to nutrient enrichment and with a large magnitude of change, though the lack of closely-related reference sequences for this organism makes it difficult to predict its role in the microbiome of genotype ML-7 or why it is lost with nutrient enrichment. The genus *Ruegeria*, which responded positively to nutrient enrichment, has been proposed to play a beneficial role in coral microbiomes by producing antimicrobial compounds that inhibit growth of *Vibrio coralliilyticus*^59^. The increase of this taxon may represent preemptive defensive activity in response to nutrient enrichment to prevent the growth of opportunistic pathogens, found to respond positively to nutrients^15,60^. The only taxon that changed under untreated conditions was the genus *Ferrimonas*, which declined slightly with time. As members of this genus are found in microbiomes of corals exposed to thermal stress^61,62^, this taxon might have been slightly elevated on the reef due to fragment collection occurring in early summer, while aquarium temperatures were maintained at 27.19 ± 0.6 °C (consistent with late spring coastal water temperatures in the Florida Keys).

### Microbiomes of a disease-resistant *Acropora cervicornis* genet do not respond to stress with dysbiosis

It has recently been proposed that the ability to tolerate significant changes in microbiome structure without negative outcomes is an important predictor in stress resistance^63^. In seven coral species exposed to white plague disease, coral species that were susceptible to disease experienced minimal microbiome changes between diseased and non-diseased states, while corals that were resistant to disease experienced much greater microbiome shifts without disease development^63^. Genotype ML-7 exhibited significant shifts in diversity over the course of this experiment in response to combined (N, P, and A) and ammonium treatments, and the relative abundance of certain key taxa was demonstrated to respond to nutrient enrichment. Yet no obvious negative outcomes were observed in response to these treatments, as growth rates for ammonium and combined treated corals were not significantly different from untreated corals by 6 weeks of exposure. Furthermore, microbiomes of genotype ML-7 did not undergo a complete community restructuring in response to nutrient enrichment despite shifts in the abundance of individual microbial members. This, coupled with previous data showing that genotype ML-7 is more resistant to white band disease development^39^, suggests that genotype ML-7 may exhibit microbiome flexibility in response to changing environmental conditions.

Microbiome flexibility has been proposed as a strategy to adapt to stress, but may risk the loss of essential symbiotic partners or allow the infiltration of opportunistic pathogens^47^. While genotype ML-50 responded to nutrient enrichment rapidly, reaching a new stable state before 3 weeks of enrichment^33^, genotype ML-7 did not exhibit significant shifts in microbiome community structure or diversity until 6 weeks of exposure to tank conditions, likely driven by the high amount of microbiome evenness exhibited by this genotype. This may suggest that while short periods of stress may be tolerated by genotype ML-7 without microbiome restructuring, sustained environmental stress may alter microbiome composition. As our nutrient exposure experiment was limited to 6 weeks, it is possible that microbial community composition in this genotype may shift further after greater periods of nutrient enrichment. Nonetheless, the same duration of exposure induced significant changes in microbial community composition in genotype ML-50^33^. In contrast to the results from the present study, a recent study including genotype ML-7 found that this genotype had reduced survival rates in response to experimental ammonium and phosphate enrichment and to a combination of nutrient enrichment and thermal stress in comparison to other genotypes harboring higher abundance of the putative parasite *Aquarickettsia*^64^. Corals in this previous study were allowed to acclimate to low-nutrient tank conditions for 4 months prior to enrichment, and this longer acclimation period to ex situ conditions may have created a greater shock upon exposure to nutrient enrichment, as no enrichment-related mortality was observed under the conditions of the present study. Furthermore, ammonium enrichment levels in the previous study were double the highest (4× ambient) concentrations used in this study. Further work is needed to elucidate the effects of nutrient enrichment on restoration genotypes after outplanting, the role the microbiome plays in regulating these effects, and whether removal or reversal of these stressors allows the microbiome to recover.

### Microbiome diversity may be a biomarker for stress tolerance

In this study, we found that the disease-resistant *Acropora cervicornis* genet “ML-7” harbored high microbial diversity and that microbiomes were largely able to withstand nutrient enrichment, experiencing few significant changes in composition or diversity. While this study was limited to the use of only one genet, only a very small number of disease-resistant *A. cervicornis* genotypes have been identified^39,40^. Indeed, only two of sixteen Florida Keys genotypes screened by Miller et al*.* in 2016 were disease resistant, and no genotype was disease resistant when fourteen different genotypes were screened in 2017^40^. An even lower prevalence of naturally disease resistant genotypes (6% of 49 genotypes) was found in Panama during wild coral surveillance^65^. As we had limited access to disease-resistant genets, we elected to instead increase replication within this genotype, with an average of ~ 5.9 replicates per treatment (other than T0, Supp. Table 2), and found consistent patterns between replicates. Disease resistant *A. cervicornis* genotypes within Mote’s nurseries exhibit consistent signatures of high microbial diversity, and are distinct in composition from *Aquarickettsia*-dominated disease susceptible genets^31,32^. We therefore contend that patterns observed in this study can be used to make predictions for microbiome responses of other disease-resistant *A. cervicornis* genets to nutrient stress.

Microbiomes of *Acropora cervicornis* genotype ML-7, previously found to be disease-resistant, were more stable in response to nutrient enrichment when compared to microbiomes of genotype ML-50, identified as disease-receptive. Results from this study parallel those from a separate study, in which exposure of individuals of genotype ML-7 to thermal stress did not significantly alter alpha diversity, community dispersion, or the abundance of any single bacterial taxon^31^. In contrast, many other genotypes of *A. cervicornis* respond to stressors by experiencing dramatic shifts in abundance of the genus *Aquarickettsia*. Bleaching of genotype ML-50 induced a near-complete loss of *Aquarickettsia*^31^, normally high abundance in this genotype. The loss of this dominant taxon allowed for the infiltration of putative pathogens including members of the order Alteromonadales, that appeared to occupy the niche space abandoned by *Aquarickettsia*. Nutrient enrichment also significantly increased abundances of *Aquarickettsia* in disease-susceptible genotypes of *A. cervicornis* with as little as 3 weeks of nutrient exposure^33^. In this study, we found that even with 6 weeks of nutrient enrichment, microbiomes of these two genotypes remained distinct, and genotype ML-7 experienced minimal changes in community structure, in contrast with genotype ML-50, which was lower diversity and more susceptible to microbiome shifts in response to nutrient enrichment. Microbiome dysbiosis, in the form of significant and rapid changes in abundance of *Aquarickettsia* with corresponding effects on microbiome structure, may therefore contribute to the disease susceptibility of genotypes including ML-50, especially as bleaching and nutrient enrichment, and their co-occurrence, becomes increasingly common^12,66^.

## Materials and methods

### Nutrient enrichment experimental design and methodology

To test the individual and combined effects of different forms of nutrient enrichment on *Acropora cervicornis* microbial community composition and coral health, a 6-week tank experiment (Supp. Fig. 1) was conducted as previously described^33^ at the Mote Marine Laboratory International Center for Coral Reef Research & Restoration (24° 39′ 41.9′′ N, 81° 27′ 15.5′′ W) in Summerland Key, Florida from April to June 2019. A total of 180 fragments (~ 5 cm) were collected from *Acropora cervicornis* genotype ML-7 (Coral Sample Registry accession: faaf3e37-60da-b78b-c753-7a0e2abd0458) from the Mote Marine Laboratory in situ coral nursery in April 2019. Fragments were originally propagated from a single donor colony, identified as genotype ML-7, and allowed to grow into new colonies in the nursery. This genotype (as delineated via multiple SNP runs (clonal ID HG0551) and microsatellite genotyping^39^) was previously found to be disease resistant^31,39^. Genotype ML-7 is known to host *Symbiodinium fitti*, the primary species of *Symbiodiniaceae* found in the Mote *A. cervicornis* nursery corals^39,67^. During this same period in April 2019, ramets of genotype ML-50 were separately exposed to identical nutrient treatment—these results are published separately due to the high sample size of the two experiments and to the unique stress responses observed in each genotype. Prior to experimental manipulation, fragments were allowed to acclimate to aquarium conditions for 7 days. Eight fragments were lost due to mortality from transplantation.

After acclimation, each aquarium was randomly assigned one of nine nutrient treatments (Supp. Fig. 1, n = 3 per treatment). Corals in aquaria were exposed to elevated levels of each nitrate (N, in the form of NaNO_3_), ammonium (A, as NH_4_Cl), phosphate (P, as Na_3_PO_4_), a combination of the three (Combined, C), or a no-treatment control. Inorganic nutrient enrichment was performed as previously described^33^ to approximately 3× or 4× ambient source water concentrations (Supp. Fig. 1). As Mote Marine Laboratory utilizes nearshore canal water as the source for its aquarium system, nitrate levels were likely higher in aquarium source water than on Looe Key Reef, from which these corals were collected. Looe Key nitrate levels as measured in the early 2010s were 3.5× lower than our nearshore source water, although as nutrient levels are rising in the Florida Keys we anticipate this discrepancy is decreasing yearly^12^. Nutrient treatments were randomly distributed across raceways. Nutrient samples were collected at 2 and 5 weeks of exposure as previously described^33^ and processed on a 3-channel AA3 HR Autoanalyzer (Seal Analytical, Southampton, UK) in Sarasota, FL.

Coral total linear extension (TLE) was assessed by measuring fragments at their longest point initially and biweekly thereafter. Due to the non-normal nature of the TLE data, data were log-transformed before a Tukey’s Honest Significance test was used to compare differences in TLE by nutrient treatment. Rough assessments of algal symbiont concentrations and coral health were made weekly using a CoralWatch Coral Health Chart^68^. The CoralWatch Coral Health Chart provides a six-point scale with which changes in coral color can be measured as an indicator of symbiont density. Photographs of each coral individual were taken contemporaneously with coral health card measurements.

### Sample collection, DNA extraction, high throughput amplicon library preparation, and sequencing

Coral fragments were sacrificed for sampling at three time points throughout the experiment: prior to nutrient exposure (T0), after 3 weeks, and after 6 weeks. Using sterile bone cutters, tissue was scraped from each fragment (avoiding the apical tip) and added directly to 2 ml tubes containing 0.5 ml DNA/RNA shield (Zymo Research) and Lysing Matrix A (MP Biomedicals, 0.5 g garnet matrix and one 1/4′′ ceramic sphere). Tubes were immediately preserved at − 80 °C until further processing. Total DNA was extracted from 500 μl of tissue slurry using the E.Z.N.A.® DNA/RNA Isolation Kit (Omega Bio-Tek) and then stored at − 80 °C until further processing. DNA yield from a total of 9 samples was insufficient for PCR (Supp. Table 1).

The V4 region of the 16S rRNA gene was amplified via 2-step polymerase chain reaction (PCR) utilizing forward and reverse primers 515F (5′-GTG YCA GCM GCC GCG GTA A-3′)^69^ and 806R (5′-GGA CTA CNV GGG TWT CTA AT-3′)^70^ and Accustart™ II PCR ToughMix (QuantaBio). Dual indices with custom adapters were used to barcode products from initial reaction in a secondary reaction (see previously published methods in^33,61,71^ for further details). Purified products were subsequently pooled in equimolar proportions based on molecular weight and DNA concentrations. Libraries were sequenced at Oregon State University’s Center for Quantitative Life Sciences (CQLS) Core Laboratories on an Illumina MiSeq sequencing platform, 2 × 300 bp version 3 chemistry according to the manufacturer’s specifications.

### Quality control and pruning of amplicon sequence data

Demultiplexing and barcode removal was performed using cutadapt^72^, during which reads with no barcode match were discarded. Quality control, sequence variant inference, and ASV table construction was performed on a total of 7,591,634 reads across 163 samples (mean depth of 46,574 reads/sample) using DADA2^73^ in R (version 4.0.3, R Development Core Team, 2019) as follows: based on quality plots, forward and reverse reads were truncated at their 3′ end at 250 and 200 base pairs, respectively. Sequences were also truncated at the first position having a quality score less than or equal to 10, and reads with a total expected error of > 2 or with the presence of Ns were discarded, resulting in a total of 3,652,086 reads. Three samples were removed that sequenced poorly, with a read depth of less than 100 reads after initial quality control (Supp. Table 1). Two sequenced PCR negatives were dominated by the genus *Anaerococcus* (order Clostridiales) and the family *Corynebacteriaceae* with an average read depth of 5254 reads.

Using DADA2, an initial total of 4452 unique amplicon sequence variants (ASVs) were inferred from unique reads and paired-end reads were subsequently merged. ASVs that did not match a target length of 252 ± 2 (127 ASVs) were discarded. Two-parent chimeras (bimeras, n = 612) were removed and taxonomy was assigned to ASVs at 100% sequence identity using the SILVA reference database (v138.1). The resulting table contained 3941 unique ASVs across 160 samples and was imported into *phyloseq* (v1.36.0^74^) for ASV annotation. A total of 288 ASVs annotated as mitochondrial, chloroplast, or eukaryotic sequences were then removed from the dataset using *phyloseq*, corresponding to a total of 1,246,196 reads. Although there were no singletons in the final dataset, 2398 ASVs were present in only one sample each. Samples with less than 1000 reads after final quality control were removed, resulting in the loss of six samples and 36 ASVs present only in these samples (Supp. Table 1). After processing in *phyloseq*, ASVs classified only to a certain taxonomic level (e.g., annotated only to the family level) were renamed at lower taxonomic levels to reflect the lowest annotated level (e.g., ASV2, unknown genus in family *Helicobacteraceae* renamed at genus level to ‘Unclassified *Helicobacteraceae* ASV2’). After processing in *phyloseq*, the Silva taxonomic classification for the genus MD3-55 was changed to *Aquarickettsia* in accordance with current identification in the literature^49^.

### Microbial diversity statistical analyses

After DADA2 quality control and removal of contaminant reads, a total of 3617 amplicon sequence variants (ASVs) remained in the dataset of 154 samples (frequency per ASV: median = 14.0, mean = 615.4, min = 2, max = 1,258,222). Observed species richness was plotted by sequencing depth using alpha rarefaction curves in *phyloseq* (Supp. Fig. 2). Alpha diversity analyses and analyses utilizing relative abundance were performed on unpruned, unrarefied data transformed to relative abundance. Differences observed in Shannon–Wiener diversity index^75^ between groups (groups exposed to different nutrient treatments, different levels of individual nutrients, and different exposure durations) were tested with the Pairwise Wilcoxon Rank Sum Test with FDR multiple test correction.

Due to the presence of numerous high diversity samples that would be compromised by rarefaction, rarefaction was not performed and instead highly rare taxa were pruned out and centered log-ratio (CLR) transformation performed to capture ratio relationships between taxa using the tool *clr* from the microbiome package^76^. ASVs with a total count across the dataset in the bottom first-quartile (count ≤ 6 across all samples) were pruned resulting in a total of 2598 unique ASVs and a median sequencing depth of 13,729 reads per sample. This equated to a removal of only 3975 reads across the dataset and 1019 ASVs. Replication by treatment and exposure weeks after quality control is detailed in Supp. Table 2. Beta diversity analyses were performed on the pruned, CLR-transformed ASV table. Principal components analysis (PCA) was performed using Euclidean distances calculated from the CLR-transformed dataset (producing Aitchison distance^77^ with the *phyloseq* command *ordinate* (as RDA without constraints).

Permutational Multivariate Analysis of Variance (PERMANOVA^78^) was performed on Euclidean distances to test for differences in beta diversity of bacterial community compositions among groups. PERMANOVA was performed using the function *adonis* from the package *vegan* (v2.5.5^79^) and was followed by pairwise analysis of variance with *pairwiseAdonis* (v0.01^80^) using Euclidean distance and 999 permutations. Permutational Analysis of Multivariate Dispersion analysis (PERMDISP^81^) was performed to examine shifts in multivariate dispersion over time and between groups using the function *betadisper*. Pairwise analysis was performed using the function *permutest* with FDR adjusted P-values (both in *vegan* v2.5.5^79^).

### Microbial differential abundance analysis

Differential abundance analysis was performed using *ANCOM-II* v2.1^82^ to identify taxa that were differentially abundant by treatment group. The unrarefied, pruned ASV table was first subset to contain only taxa with at least 20 counts in at least 10% of samples and summarized to the genus level with the *tax_glom* command in *phyloseq*. This resulted in a table with 35 genera within 154 samples with a median sequencing depth of 11,804 reads per sample. One model was run for each of several pairwise contrasts between combinations: Untreated samples vs T0, nutrient (all types of nutrient treatment) vs T0, and each individual treatment at 3 and 6 weeks compared to T0, and 6 vs 3 weeks. Tank identity was included in each model as a random effect.

### Genotypic comparisons based on identical nutrient treatment

A comparative analysis was also conducted comparing the samples of *Acropora cervicornis* genotype ML-7 (this study) with 150 samples of genotype ML-50 (Coral Sample Registry accession: fa13971c-ea34-459e-2f13-7bfddbafd327)^33^, also exposed to nutrient enrichment for 6 weeks under the same parameters and sequenced on the same Illumina MiSeq run. A combined total of 14,215,265 reads in 304 samples (154 of genotype ML-7, 150 of genotype ML-50) were processed together using DADA2 and taxonomic assignment and quality control was conducted as described above. A total of 4396 ASVs remained after removal of chimeras and mitochondrial, chloroplast, and eukaryotic contamination. ASVs with a total count across the dataset in the bottom first-quartile (count ≤ 5) were removed resulting in a final total of 5,149,563 total reads including 3153 unique ASVs. Median read depth per sample was 16,408 reads. ANCOM was used to identify taxa that were differentially abundant by genotype at the beginning and end of the experiment. For this, the ASV table was first filtered to contain only taxa with at least 10 counts in 10% of samples and summarized to the genus level with the *tax_glom* command in *phyloseq*. This resulted in a table with 35 genera within 304 samples with a median sequencing depth of 16,436 reads per sample. For comparisons of genotypes ML-50 and ML-7, we selected a more conservative significance threshold of W = 0.8 and performed comparisons by genotype at T0 and at week 6. Tank identity was included in each model as a random effect.

## Supplementary Information

Supplementary Information.

## Data Availability

Raw sequence data were deposited into the NCBI Sequence Read Archive (SRA) under BioProject PRJNA756691. All scripts involved in the analysis of this dataset are available at https://github.com/graceklinges/disease-resistant-micro. Intermediate datasets generated during analysis of samples from the current study are available from the corresponding author on request.
