# Supplementary material for: Microbiomes of a disease-resistant genotype of Acropora cervicornis are resistant to acute, but not chronic, nutrient enrichment
Source: Sci Rep. 2023 Mar 3;13:3617. doi: 10.1038/s41598-023-30615-x (PMC9984465; doi:10.1038/s41598-023-30615-x)
Supplement: Supplementary file 1 — Supplementary Information. [file 41598_2023_30615_MOESM1_ESM.pdf]

Supplemental Figure Legends

Supplemental Figure 1. Experimental design for nutrient enrichment experiment. Three replicate tanks were used per treatment, with two levels of enrichment (3x and 4x ambient, L and H respectively) for each nutrient constituent. Each tank contained five coral fragments, with one fragment sampled per timepoint. Extra tanks were added that received no treatment to ensure sufficient numbers of ‘control’ tanks survived throughout the experiment. Although not shown in this simplified version of our experimental design, treatment tanks were randomly distributed between two raceways. The lower panel presents levels of nutrient enrichment received per treatment, as assessed by AutoAnalyzer at Mote Marine Laboratory (Sarasota, FL) Nitrogen was measured as both dissolved ammonia and nitrate-nitrite, phosphorus was measured as dissolved orthophosphate. All data are presented in micromolar concentrations.

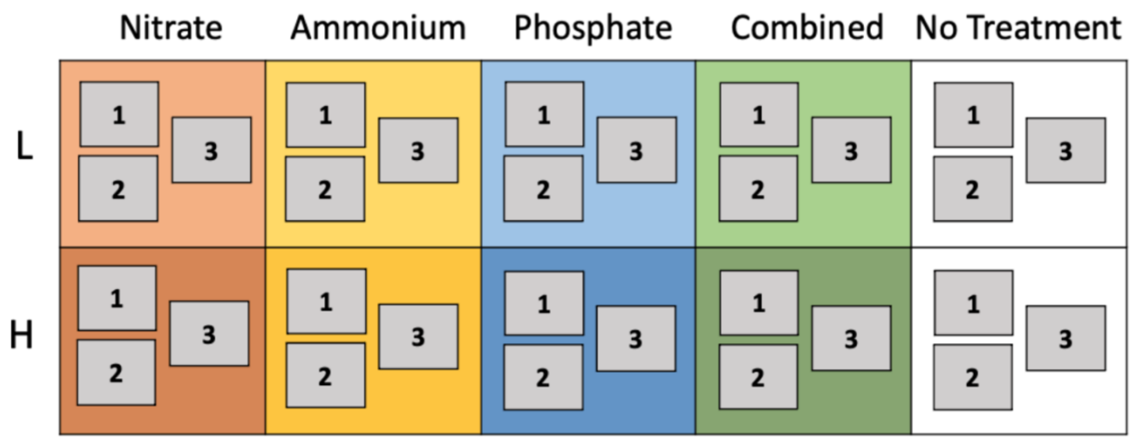

Treatment	Nitrogen, Nitrate-Nitrite (as N), Diss. μM	Nitrogen, Ammonia (as N), Diss. μM	Phosphorus, Orthophosphate (as P), Diss. μM
**L Ammonium**	2.06 ± 0.047	**3.78 ± 0.66**	0.115 ± 0.020
**H Ammonium**	2.17 ± 0.307	**5.15 ± 1.75**	0.115 ± 0.012
**L Nitrate**	**8.69 ± 1.38**	0.829 ± 0.144	0.116 ± 0.017
**H Nitrate**	**12.1 ± 1.90**	0.886 ± 0.142	0.129 ± 0.015
**L Phosphate**	1.98 ± 0.116	0.696 ± 0.268	**0.431 ± 0.035**
**H Phosphate**	2.12 ± 0.242	0.848 ± 0.188	**0.607 ± 0.240**
**L Combined**	**9.01 ± 0.90**	**3.99 ± 0.41**	**0.440 ± 0.041**
**H Combined**	**12.6 ± 5.22**	**5.81 ± 2.34**	**0.620 ± 0.253**
**No Treatment**	2.04 ± 0.058	0.81 ± 0.13	0.118 ± 0.013
**Looe Key Reef, 2010s (Lapointe et al., 2019)**	0.55 ± 0.05	0.57 ± 0.04	0.11 ± 0.01

Supplemental Figure 2. Alpha rarefaction curves produced by plotting observed species richness by sequencing depth using alpha rarefaction curves in *phyloseq*. Dashed line represents the minimum sequencing depth in the dataset. Rarefaction was not performed for any analysis because of the high number of ASVs that would be lost through rarefaction and the subsequent deflation of diversity that would be induced through this transformation.

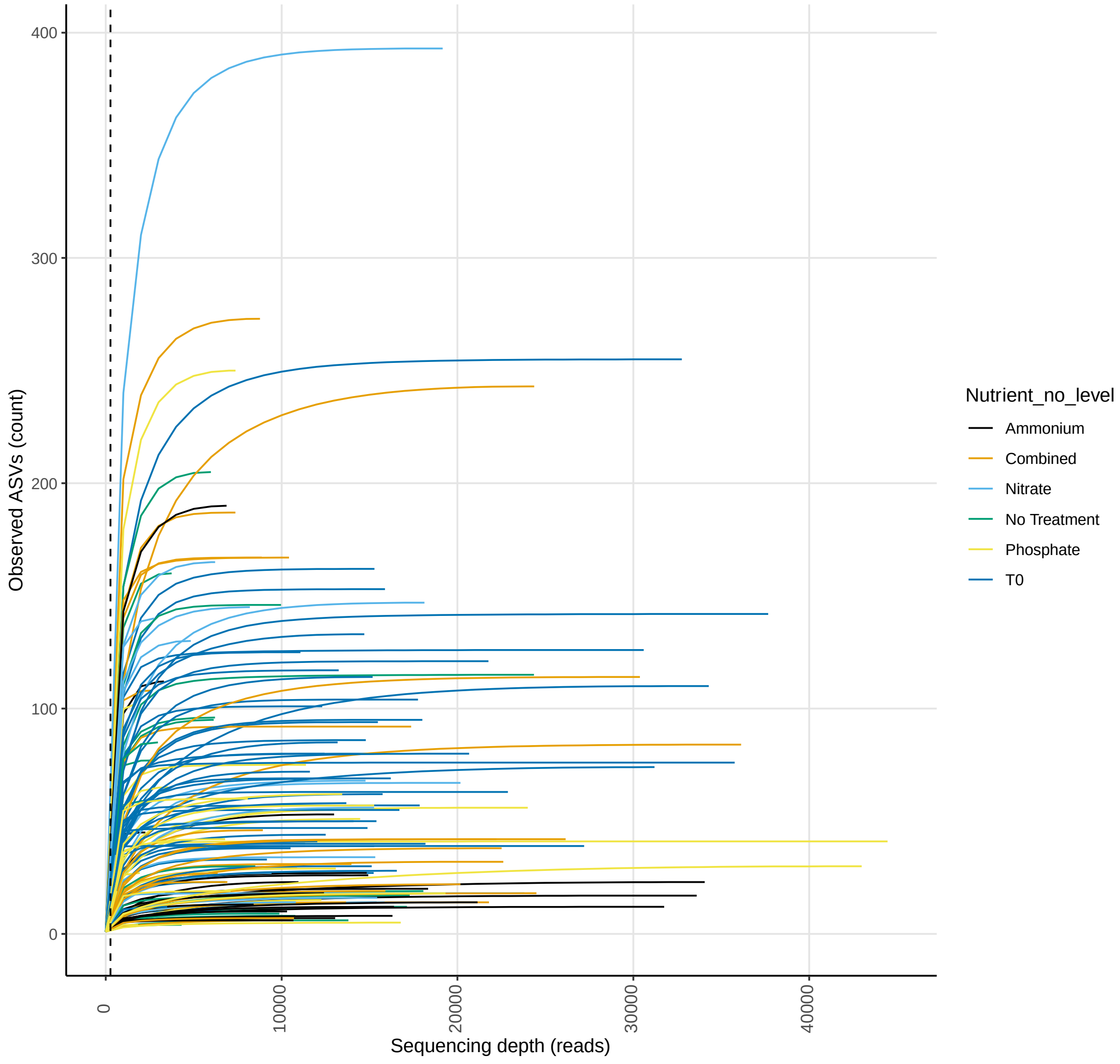

Supplemental Figure 3. Differences in Shannon's index of diversity by treatment group: Nutrient (Ammonium, Combined, Nitrate, Phosphate) vs. No Treatment, and exposure weeks (0, 3, and 6). Boxes sharing a letter are not significantly different from each other using an FDR corrected significance level of $p < 0.05$.

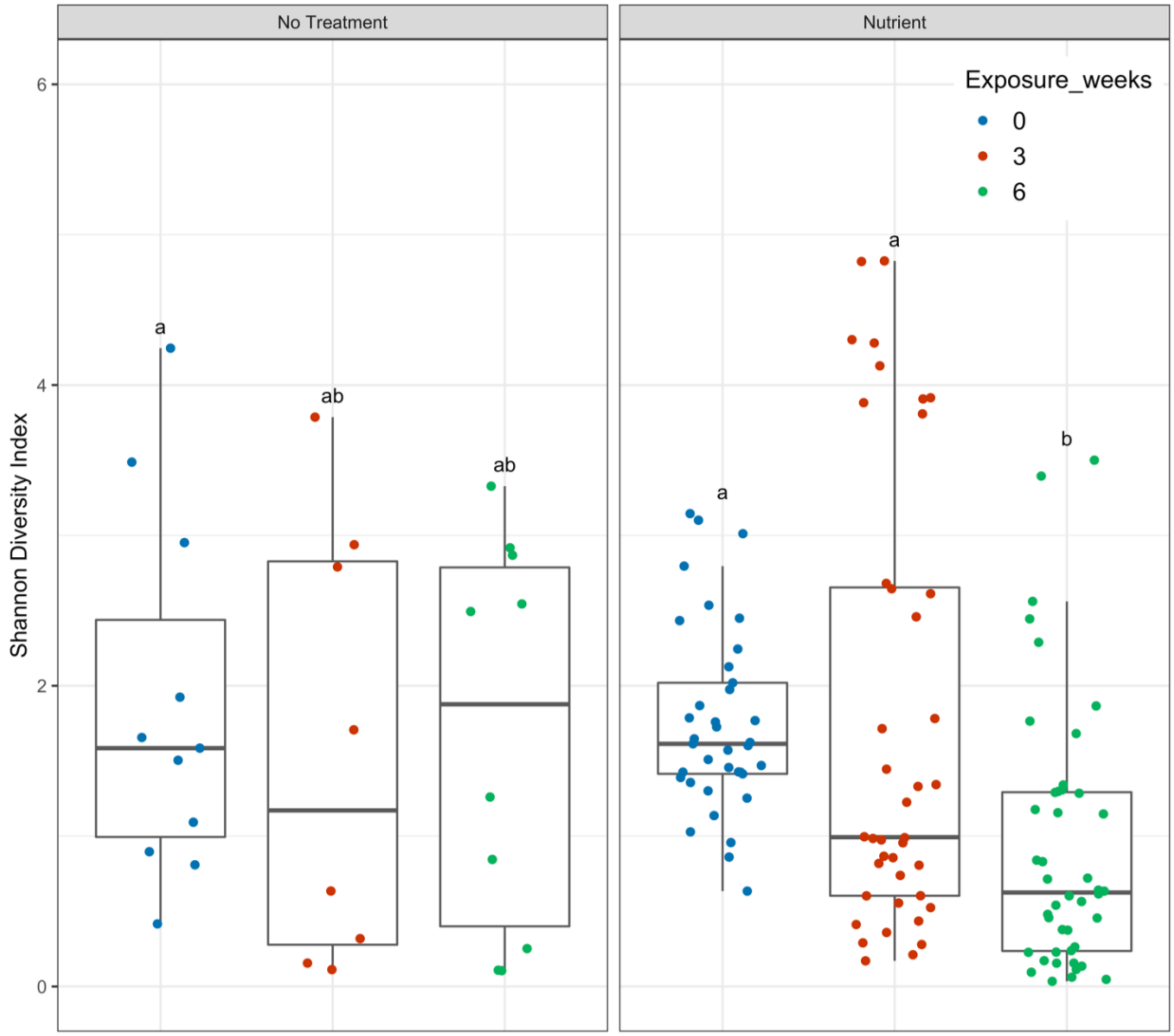

Supplemental Figure 4. Differences in dispersion, as distance-to-centroid, by treatment (Ammonium, Combined, Nitrate, Phosphate, or no treatment) and exposure weeks (0, 3, and 6) in genotype ML-7. Boxes sharing a letter are not significantly different from each other using an FDR corrected significance level of $p < 0.05$.
significance level of $p < 0.05$.

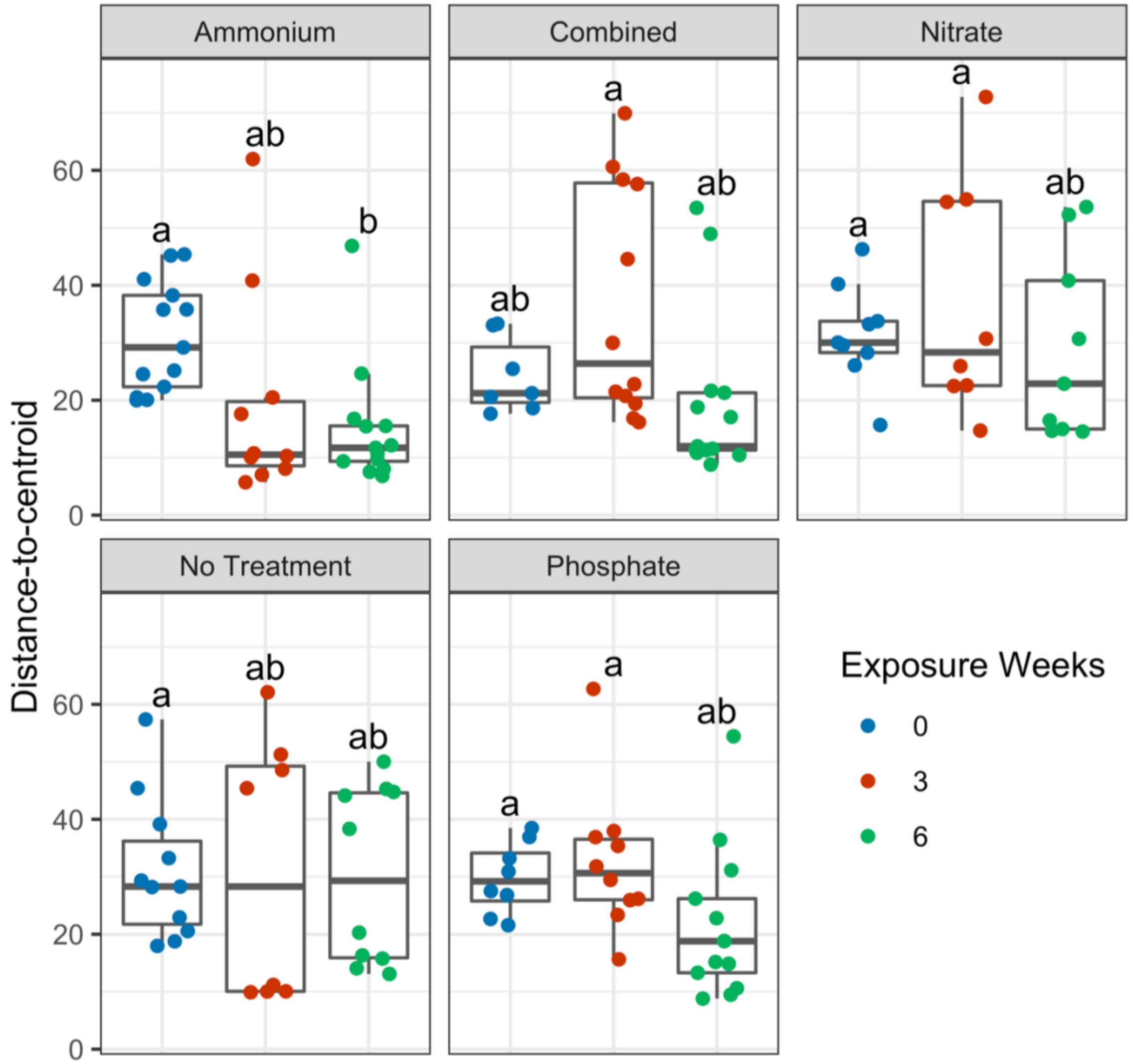

Supplemental Figure 5. Volcano plot of results from differential abundance analysis with ANCOM-II on genotype ML-7 samples by treatment condition at 6 weeks vs 0 weeks: Expanded version of Figure 4. Data were subset to either only no treatment samples, or all other treatments pooled together (“nutrient”). The W statistic represents the strength of the test for the 35 tested species. Taxa above the dashed line are significant with the null-hypothesis rejected 70% of the time (W = 0.7). Non-significant taxa are colored grey. The x-axis value presents the effect size as the CLR (centered log ratio)-transformed mean difference in abundance of a given species between the two groups being compared. A positive x-axis value indicates that a genus was more abundant at 6 weeks than 0 weeks (abundance increased with time)

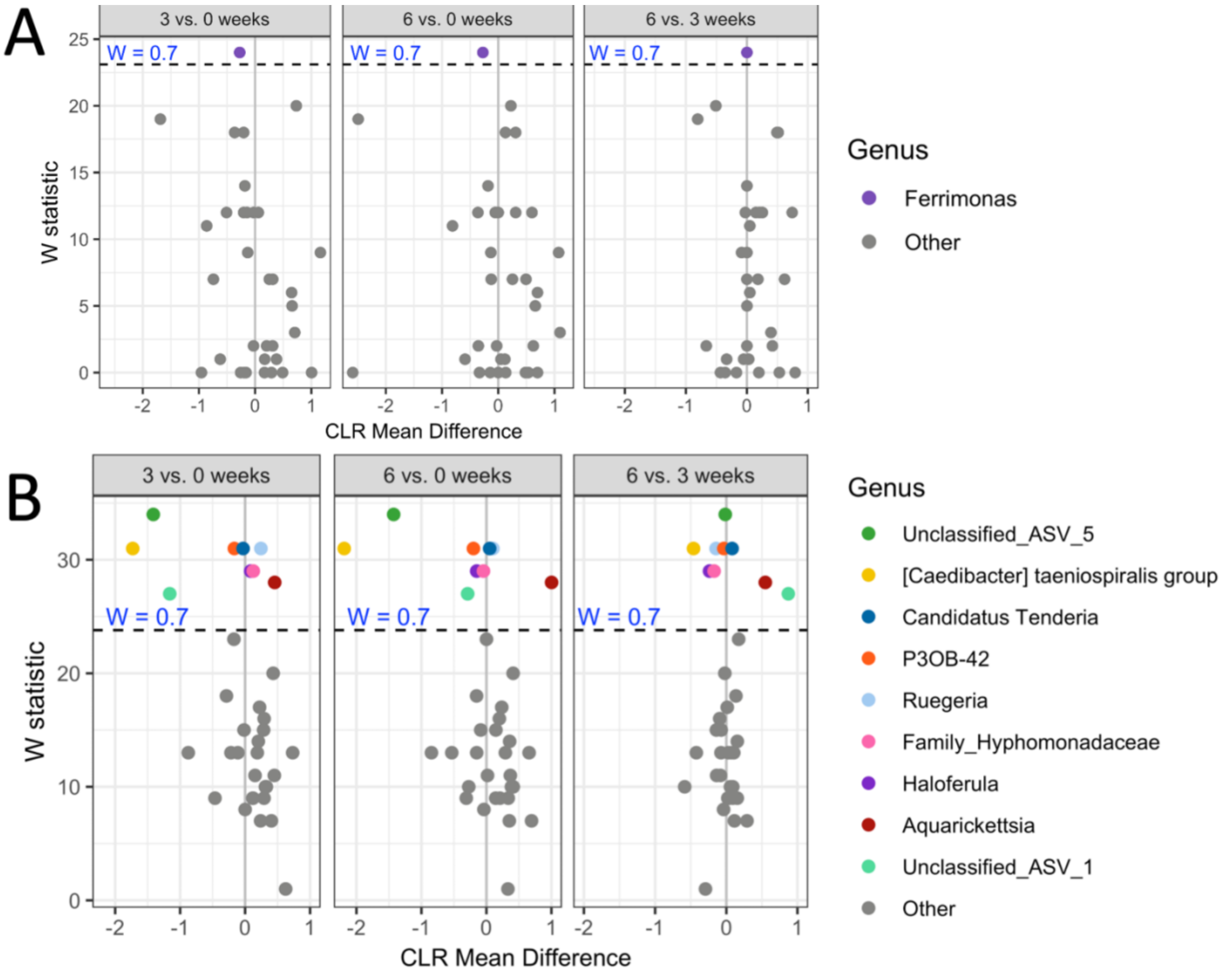

Supplemental Figure 6. Total linear extension (TLE) calculated from measurements taken at 0, 3, and 6 weeks of nutrient exposure. Tukey's honest significance test was used on log-transformed data to identify significant differences in mean TLE by nutrient treatment and exposure weeks. Untransformed data is plotted for clarity. Boxes sharing a letter are not significantly different from each other using a significance level of $p < .05$.

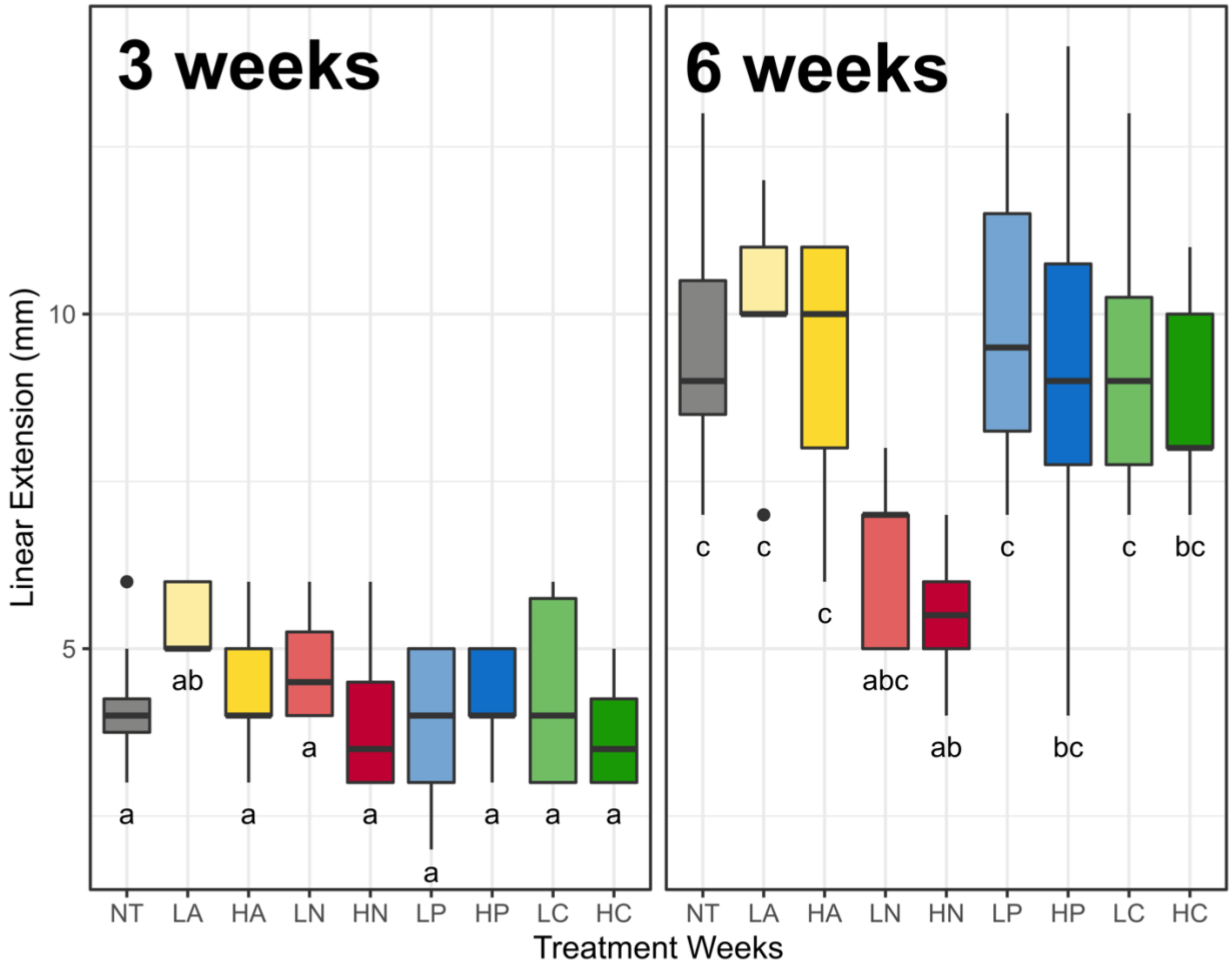

Supplemental Figure 7. Differences in Shannon's index of diversity by genotype (ML-50 and ML-7) exposure weeks (0, 3, and 6). Boxes sharing a letter are not significantly different from each other using an FDR corrected significance level of $p < 0.05$.

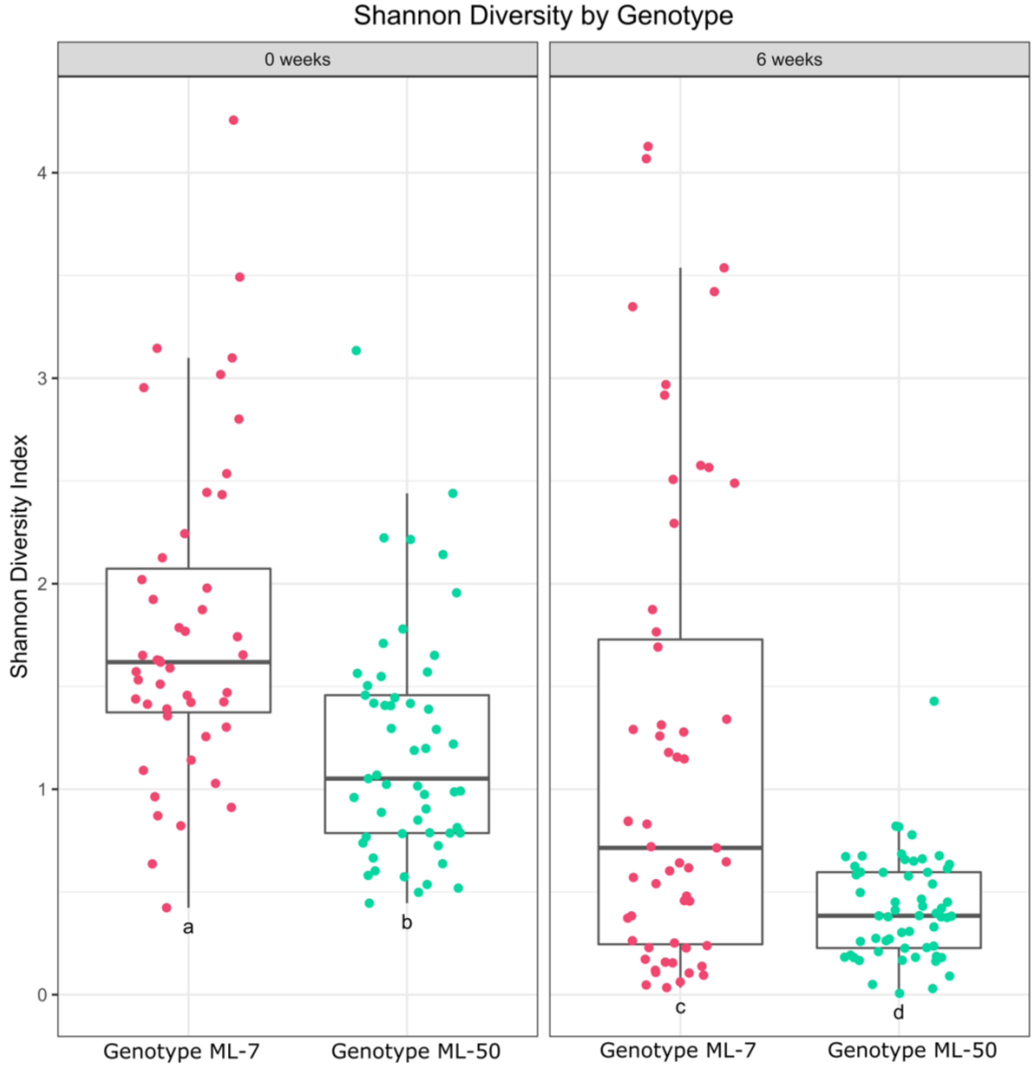

Supplemental Figure 8. Principal components analysis ordination of genotype ML-7 and genotype ML-50 nutrient-treated samples using Euclidean distance on centered log ratio-transformed data, colored by genotype (**A**) and nutrient treatment (**B**).

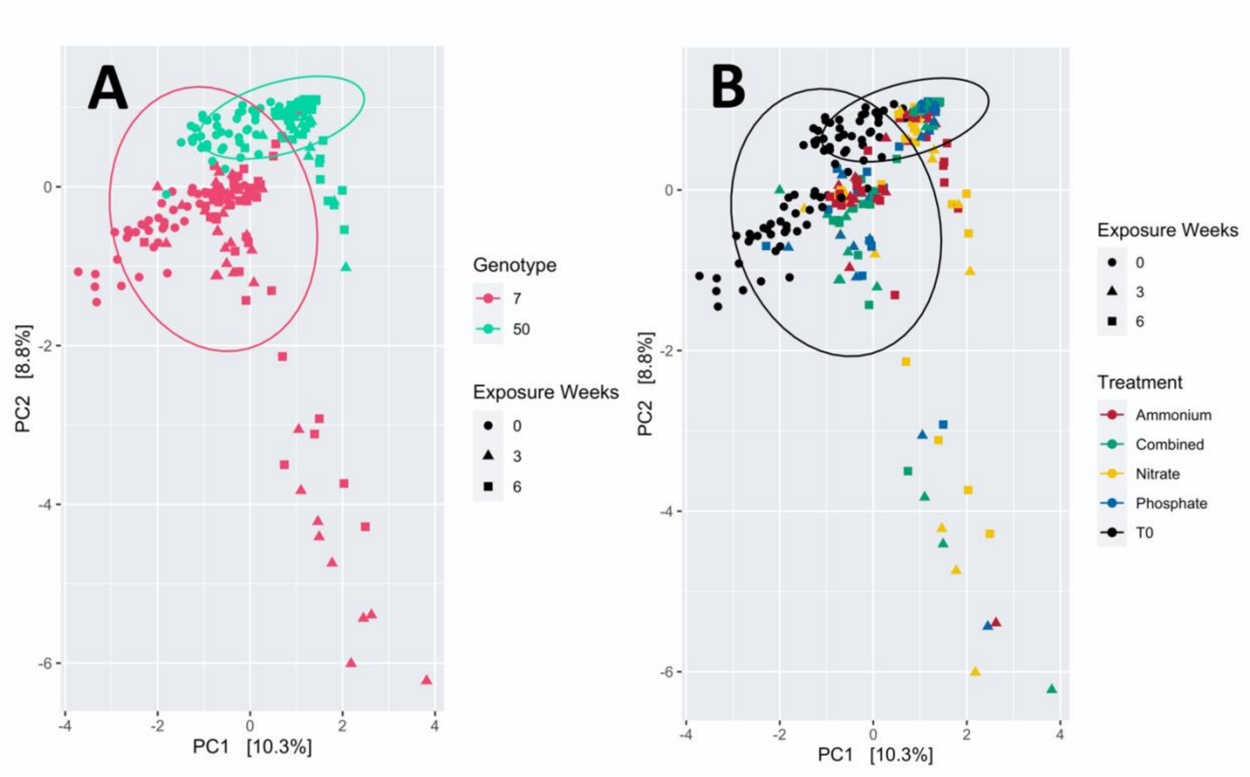

Supplemental Figure 9. Differences in dispersion, as distance-to-centroid, by genotype (ML-50 and ML-7) and exposure weeks (0, 3, and 6) in nutrient-treated samples. Boxes sharing a letter are not significantly different from each other using an FDR corrected significance level of $p < 0.05$.

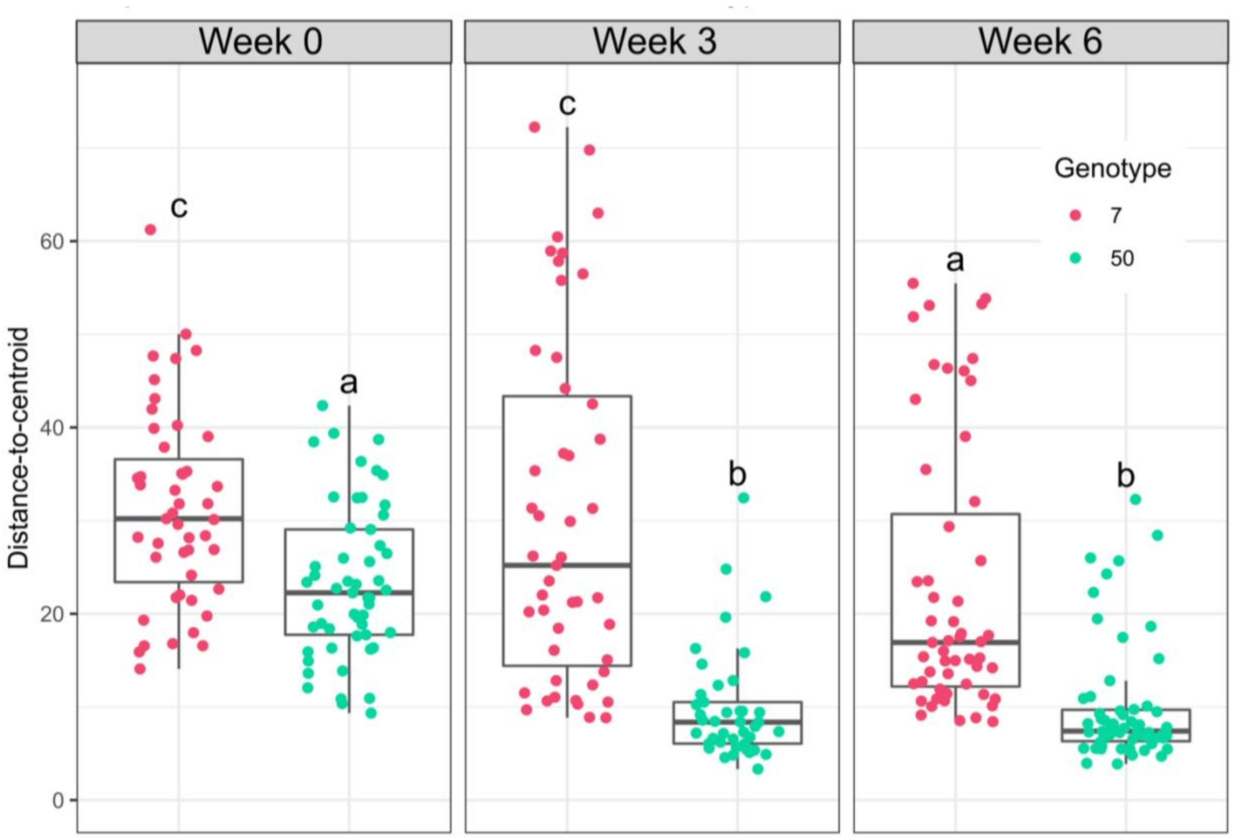

Supp. Table 1. Samples lost throughout processing and analysis.

Reason for Loss	Number Lost	Sample IDs
Lost to transplantation/ acclimatization:	8	Sample IDs had not been assigned yet
Low DNA yield:	9	LA-7-1-T1, HC-7-3-T3, CTRL-7-2-T3, CTRL-7-1-T1, LC-7-1-T1, LP-7-2-T3, HP-7-2-T2, LA-7-1-T2, MC-7-1-T2
Less than 100 reads after initial QC	3	MN-7-1-T2, LP-7-2-T2, HA-7-6-TB
Less than 1000 reads after final QC	6	CTRL-7-3-T4, HN-7-2-T3, LA-7-1-T4, MN-7-1-T3, MN-7-1-T4, MP-7-1-T3

Supp. Table 2. Final sample replication by treatment.

Group	Treatment	Time	Samples
**T0**	**T0**	Week 0	48
**Nitrate T3 = 8 T6= 9**	**LN**	Week 3	3
		Week 6	4
	**HN**	Week 3	5
		Week 6	5
**Ammonium T3 = 10 T6 = 13**	**LA**	Week 3	5
		Week 6	6
	**HA**	Week 3	5
		Week 6	7
**Phosphate T3 = 10 T6= 13**	**LP**	Week 3	5
		Week 6	5
	**HP**	Week 3	5
		Week 6	8
**Combined T3 = 12 T6= 13**	**LC**	Week 3	6
		Week 6	8
	**HC**	Week 3	6
		Week 6	5
**Untreated**	**NT**	Week 3	8
		Week 6	10
		**Total**	**154**

Supp. Table 3. P values associated with Pairwise Wilcoxon Rank Sum Test with FDR correction for tests for differences in Simpson's diversity between individual treatments. A = ammonium, C = combined, N = nitrate, NT = no treatment, P = phosphate. 0, 3, 6 refer to zero, three, and six weeks of experimental conditions.

	A0	A3	A6	C0	C3	C6	N0	N3	N6	NT0	NT3	NT6	P0	P3
**A3**	0.082	NA	NA	NA	NA	NA	NA	NA	NA	NA	NA	NA	NA	NA

**A6**	0.017	1.000	NA	NA	NA	NA	NA	NA	NA	NA	NA	NA	NA	NA
**C0**	1.000	0.187	0.082	NA	NA	NA	NA	NA	NA	NA	NA	NA	NA	NA
**C3**	0.857	0.109	0.082	1.000	NA	NA	NA	NA	NA	NA	NA	NA	NA	NA
**C6**	0.006	0.833	0.675	0.028	0.021	NA	NA	NA	NA	NA	NA	NA	NA	NA
**N0**	0.918	0.089	0.025	0.897	0.834	0.007	NA	NA	NA	NA	NA	NA	NA	NA
**N3**	1.000	0.133	0.095	1.000	0.925	0.025	0.925	NA	NA	NA	NA	NA	NA	NA
**N6**	0.739	0.389	0.180	0.834	1.000	0.082	0.661	0.834	NA	NA	NA	NA	NA	NA
**NT0**	1.000	0.109	0.059	1.000	1.000	0.015	0.918	1.000	0.864	NA	NA	NA	NA	NA
**NT3**	0.834	0.918	0.834	1.000	0.729	0.346	0.834	0.680	1.000	0.834	NA	NA	NA	NA
**NT6**	1.000	0.834	0.419	1.000	0.857	0.154	0.969	0.834	1.000	0.897	1.000	NA	NA	NA
**P0**	1.000	0.133	0.040	1.000	0.871	0.015	0.834	0.918	0.827	1.000	1.000	1.000	NA	NA
**P3**	1.000	0.082	0.031	1.000	0.834	0.007	0.834	1.000	0.834	1.000	0.845	1.000	1.000	NA
**P6**	0.089	0.834	0.730	0.230	0.546	0.287	0.076	0.478	0.554	0.269	0.871	0.834	0.112	0.133

Supp. Table 4. P values associated with pairwise PERMANOVA with FDR correction for tests for differences in community composition between individual treatments. A = ammonium, C = combined, N = nitrate, NT = no treatment, P = phosphate. 0, 3, 6 refer to zero, three, and six weeks of experimental conditions. Pairwise comparisons discussed in the manuscript text are bolded. Other significant comparisons (not bolded) were comparisons of different treatments between timepoints, and therefore not biologically relevant.

**pairs**	**F.Model**	**R2**	**p.value**	**p.adjusted**
**T0 vs C3**	3.21771931	0.05256189	0.001	0.005
**T0 vs N3**	3.22929467	0.0564273	0.001	0.005
**T0 vs P3**	2.83410983	0.0481712	0.002	0.00846154
**T0 vs A3**	2.49465849	0.04264763	0.001	0.005
**T0 vs N6**	3.77810575	0.06427743	0.001	0.005
**T0 vs NT3**	2.64686719	0.04672575	0.001	0.005
**T0 vs NT6**	3.42105159	0.05757306	0.001	0.005
**T0 vs C6**	3.17620839	0.05108398	0.001	0.005
**T0 vs P6**	2.38002148	0.03877518	0.001	0.005
**T0 vs A6**	3.45074652	0.05525549	0.001	0.005
C3 vs N3	0.83553131	0.04435932	0.811	0.82601852
C3 vs P3	0.79660129	0.0383044	0.974	0.974
C3 vs A3	0.94731489	0.04522369	0.506	0.59212766
C3 vs N6	1.21804305	0.06024535	0.146	0.21851351
C3 vs NT3	0.87422654	0.04631854	0.779	0.80839623
C3 vs NT6	1.13521597	0.05371206	0.168	0.23073171
C3 vs C6	1.24288839	0.05126816	0.097	0.18333333
C3 vs P6	1.21283577	0.05009061	0.092	0.18071429
C3 vs A6	1.76776923	0.07137378	0.001	0.005

N3 vs P3	1.02112787	0.05999179	0.325	0.40875
N3 vs A3	1.22738323	0.07124606	0.172	0.23073171
N3 vs N6	1.25721318	0.07733264	0.141	0.21851351
N3 vs NT3	0.95235738	0.06369279	0.495	0.59184783
N3 vs NT6	1.2089834	0.07025304	0.13	0.21666667
N3 vs C6	1.69046789	0.08170274	0.029	0.08394737
N3 vs P6	1.60861272	0.07805536	0.036	0.09428571
N3 vs A6	2.36270143	0.11059938	0.004	0.01571429
P3 vs A3	0.96716129	0.05099136	0.421	0.51455556
P3 vs N6	1.2257527	0.06725389	0.156	0.22578947
P3 vs NT3	0.89431038	0.0529356	0.765	0.80839623
P3 vs NT6	1.09347446	0.05726954	0.247	0.32345238
P3 vs C6	1.21870545	0.05485043	0.075	0.15865385
P3 vs P6	1.23766042	0.05565605	0.071	0.1562
P3 vs A6	1.85636609	0.08121878	0.002	0.00846154
A3 vs N6	1.37707926	0.07493461	0.114	0.20225806
A3 vs NT3	0.91860411	0.0542955	0.573	0.6391
A3 vs NT6	1.39599038	0.07197314	0.08	0.16296296
A3 vs C6	0.91414153	0.04171469	0.658	0.70960784
A3 vs P6	0.92138072	0.04203114	0.581	0.6391
A3 vs A6	1.20373919	0.05421335	0.129	0.21666667
N6 vs NT3	1.26979632	0.07804623	0.1	0.18333333
N6 vs NT6	1.00087349	0.05560138	0.327	0.40875
N6 vs C6	1.79457847	0.08234059	0.018	0.055
**N6 vs P6**	1.86692655	0.08537672	0.012	0.03882353
**N6 vs A6**	2.52386057	0.11205275	0.008	0.02933333
NT3 vs NT6	1.1477985	0.06693562	0.163	0.22987179
NT3 vs C6	1.18114002	0.05852692	0.147	0.21851351
NT3 vs P6	1.167878	0.05790783	0.141	0.21851351
NT3 vs A6	1.74585258	0.08415429	0.009	0.0309375
NT6 vs C6	1.48127881	0.06588944	0.035	0.09428571
NT6 vs P6	1.3773397	0.06155065	0.051	0.12195652
**NT6 vs A6**	2.36605236	0.10126025	0.001	0.005
C6 vs P6	0.96089148	0.03849588	0.57	0.6391
C6 vs A6	1.28084728	0.05066473	0.049	0.12195652
P6 vs A6	1.33681105	0.05276161	0.055	0.12604167

Supplementary Table 5. P values associated with pairwise PERMANOVA with FDR correction for tests for differences in community composition between levels of nutrient exposure. 0 = no

nutrient enrichment. L = 3x ambient nutrient concentrations, H = 4x ambient nutrient concentrations.

pairs	F.Model	R2	p.value	p.adjusted
0 vs H	3.4789250	0.03177712	0.001	0.0015
0 vs L	3.7054977	0.03258855	0.001	0.0015
H vs L	0.9810697	0.01127912	0.422	0.4220

Supplementary Table 6. P values associated with pairwise PERMDISP with FDR correction for tests in differences in dispersion between treated samples (weeks three and six) and T0 samples (unexposed samples at the start of the experiment).

Pairs	p.value
T0-No Treatment	0.872
T0-Nitrate	0.669
T0-Phosphate	0.50571429
T0-Ammonium	0.015
T0-Combined	0.76772727
No Treatment-Nitrate	0.80678571
No Treatment-Phosphate	0.669
No Treatment-Ammonium	0.055
No Treatment-Combined	0.80678571
Nitrate-Phosphate	0.50571429
Nitrate-Ammonium	0.0225
Nitrate-Combined	0.669
Phosphate-Ammonium	0.084
Phosphate-Combined	0.80678571
Ammonium-Combined	0.084

Supplementary Table 7. Output of Tukey's pairwise honest significance test on log-transformed linear extension data, with comparisons performed within timepoints (3 weeks and 6 weeks).

3 weeks	p adj		6 Weeks	p adj
LA3-NT3	0.68793321		LA6-NT6	0.99999369
HA3-NT3	0.9999845		HA6-NT6	0.99999979
LN3-NT3	0.99244553		LN6-NT6	0.28157133
HN3-NT3	0.99999547		HN6-NT6	0.02464417
LP3-NT3	0.99323404		LP6-NT6	0.99999993
HP3-NT3	0.99999997		HP6-NT6	0.99506515
LC3-NT3	1		LC6-NT6	0.99999991
HC3-NT3	0.99966392		HC6-NT6	0.99996779

HA3-LA3	0.93323237		HA6-LA6	0.99988647
LN3-LA3	0.99829106		LN6-LA6	0.31759534
HN3-LA3	0.67130015		HN6-LA6	0.04956128
LP3-LA3	0.31437173		LP6-LA6	0.99999999
HP3-LA3	0.86530924		HP6-LA6	0.98369854
LC3-LA3	0.79359066		LC6-LA6	0.99990578
HC3-LA3	0.52647045		HC6-LA6	0.99916983
LN3-HA3	0.99989598		LN6-HA6	0.50319914
HN3-HA3	0.99938865		HN6-HA6	0.08866295
LP3-HA3	0.966089		LP6-HA6	0.9999886
HP3-HA3	0.99999993		HP6-HA6	0.99977162
LC3-HA3	0.99999849		LC6-HA6	1
HC3-HA3	0.99461912		HC6-HA6	0.99999988
HN3-LN3	0.97811228		HN6-LN6	0.99757008
LP3-LN3	0.82489414		LP6-LN6	0.33764631
HP3-LN3	0.99894681		HP6-LN6	0.78272516
LC3-LN3	0.99708936		LC6-LN6	0.45036756
HC3-LN3	0.93650524		HC6-LN6	0.72701032
LP3-HN3	0.99994701		LP6-HN6	0.0484541
HP3-HN3	0.99995294		HP6-HN6	0.22407615
LC3-HN3	0.99998482		LC6-HN6	0.0668066
HC3-HN3	0.99999983		HC6-HN6	0.22848822
HP3-LP3	0.98912775		HP6-LP6	0.99192004
LC3-LP3	0.99185255		LC6-LP6	0.99999191
HC3-LP3	0.99999977		HC6-LP6	0.99979781
LC3-HP3	1		LC6-HP6	0.99957164
HC3-HP3	0.99898519		HC6-HP6	0.99999848
HC3-LC3	0.99943337		HC6-LC6	0.99999965

Supplementary Table 8. P values associated with pairwise PERMANOVA with FDR correction for tests for differences in community composition between two genotypes at 0, 3, and 6 weeks of any type of nutrient enrichment.

pairs	F.Model	R2	p.value	p.adjusted
50_0 vs 7_0	7.03412099	0.08272116	0.001	0.00115385
50_0 vs 7_3	6.87360146	0.07734132	0.001	0.00115385
50_0 vs 7_6	10.1374578	0.10225658	0.001	0.00115385
50_0 vs 50_3	10.3352881	0.11834035	0.001	0.00115385
50_0 vs 50_6	14.7303697	0.1342415	0.001	0.00115385
7_0 vs 7_3	4.5797156	0.05828114	0.001	0.00115385

7_0 vs 7_6	6.5949634	0.0752893	0.001	0.00115385
7_0 vs 50_3	16.4454307	0.19246706	0.001	0.00115385
7_0 vs 50_6	23.4587301	0.21237552	0.001	0.00115385
7_3 vs 7_6	1.92128754	0.02210376	0.006	0.00642857
7_3 vs 50_3	6.67995649	0.08383484	0.001	0.00115385
7_3 vs 50_6	9.90465139	0.09815852	0.001	0.00115385
7_6 vs 50_3	9.7722523	0.10885604	0.001	0.00115385
7_6 vs 50_6	13.3745738	0.12008642	0.001	0.00115385
50_3 vs 50_6	1.16247791	0.01333691	0.203	0.203

Supplementary Table 9. P values associated with pairwise PERMDISP with FDR correction for tests in differences in dispersion between genotypes ML-50 and ML-7 at each week of the experiment.

**pair**	**p.adj**
50_0-50_3	0.00166667
50_0-50_6	0.00166667
50_0-7_0	0.00166667
50_0-7_3	0.003
50_0-7_6	0.96
50_3-50_6	0.70178571
50_3-7_0	0.00166667
50_3-7_3	0.00166667
50_3-7_6	0.00166667
50_6-7_0	0.00166667
50_6-7_3	0.00166667
50_6-7_6	0.00166667
7_0-7_3	0.69230769
7_0-7_6	0.00375
7_3-7_6	0.00375
